# Conceptual analysis of the combined effects of vaccination, therapeutic actions, and human subjection to physical constraint in reducing the prevalence of COVID-19 using the homotopy perturbation method

**DOI:** 10.1186/s43088-023-00343-2

**Published:** 2023-01-19

**Authors:** Mutairu Kayode Kolawole, Morufu Oyedunsi Olayiwola, Adedapo Ismaila Alaje, Hammed Ololade Adekunle, Kazeem Abidoye Odeyemi

**Affiliations:** Department of Mathematical Sciences, Osun State University, Osogbo, Nigeria

**Keywords:** COVID-19, Vaccination, Therapeutic action, Contact rate, Homotopy perturbation method

## Abstract

**Background:**

The COVID-19 pandemic has put the world's survival in jeopardy. Although the virus has been contained in certain parts of the world after causing so much grief, the risk of it emerging in the future should not be overlooked because its existence cannot be shown to be completely eradicated.

**Results:**

This study investigates the impact of vaccination, therapeutic actions, and compliance rate of individuals to physical limitations in a newly developed SEIQR mathematical model of COVID-19. A qualitative investigation was conducted on the mathematical model, which included validating its positivity, existence, uniqueness, and boundedness. The disease-free and endemic equilibria were found, and the basic reproduction number was derived and utilized to examine the mathematical model's local and global stability. The mathematical model's sensitivity index was calculated equally, and the homotopy perturbation method was utilized to derive the estimated result of each compartment of the model. Numerical simulation carried out using Maple 18 software reveals that the COVID-19 virus's prevalence might be lowered if the actions proposed in this study are applied.

**Conclusion:**

It is the collective responsibility of all individuals to fight for the survival of the human race against COVID-19. We urged that all persons, including the government, researchers, and health-care personnel, use the findings of this research to remove the presence of the dangerous COVID-19 virus.

## Background

COVID-19 infection has spread to every continent. New coronavirus hotspots were detected in Wuhan, China, in December 2019. When numerous people were admitted to the hospital in late December 2019, it was clear that the pandemic had begun. By mid-July 2020, the virus had infected over 213 countries, causing 15,969,465 infections and 643,390 fatalities [1]. The WHO discovered that the virus may be breathed in through normal breathing, resulting in new infections. COVID-19 has a 2 to 14 days of incubation period, with about 97.5 percent of infected individuals presenting symptoms 11 days after infection [2–4].

Several researchers and scientists have published significant and successful research works to aid in the eradication and control of COVID-19. Among these is the study reported in [5], in which the fear impact of media is divided into two categories, namely the fear of infection toll and the fear of death toll, and investigated using a proposed mathematical model. Additionally, the influence of the convex incidence rate on multiple COVID-19 transmissions was explored in [6]. According to their findings, the double exposure of vulnerable individuals can result in projected occurrences of COVID-19 infection in the general population. A novel assessment of the COVID-19 transmission dynamics was reported in [7]. The model looked at the effects of the convex incidence rate. To stabilize the pace of disease reduction in their system, a variety of strategies were utilized. Eventually, the dynamics of the disease are greatly impacted as huge drops in infection rates are observed when the rate of immigration and population mixing in the afflicted area are taken into account.

Vaccination, therapy, and human compliance to restriction of physical interactions are crucial in avoiding illness development associated with COVID-19. Several researchers have developed mathematical models to solely evaluate the impact of each of these factors in eradicating the spread of COVID-19. The results of a two-stage COVID-19 vaccination research were published in [8]. According to their findings, well-implemented immunization programs can prevent the spread of COVID-19. In [9], research was done on the SEIRS model for COVID-19 that captured saturation incidence and treatment response for the global study. In their investigation, they came to the conclusion that, even if treatment can reduce the spread of COVID-19, lowering the effective contact rate is the best way for people and the government to do so. Additionally, a mathematical model that can evaluate the effect of mask use on the general populace was proposed in [10], and a SIR COVID-19 pandemic model was investigated using statistical methods in [11].

The course of diseases may usually be accurately and realistically predicted by performing a numerical simulation of epidemic models. To forecast the COVID-19 virus's future existence, a mathematical model of the virus was built, investigated, and simulated in [12] using actual data from Pakistan. Researchers in [13] looked at the logistic growth model that might be used to estimate the scope of the COVID-19 epidemic. In [14], a mathematical analysis of a stochastic model for coronavirus propagation was reported. A collocation approach based on Legendre polynomials was used to achieve the numerical solution of this system, and simulations were used to review the results and pandemic model findings, and a recent research presented in [15] features the simulation of fractional-order Caputo’s derivative on a coronavirus disease model using the Laplace–Adomian decomposition method.

Since these mathematical models are typically nonlinear, strong numerical techniques, such as the homotopy perturbation approach offered by [16], are often required to compute their approximate solution. This homotopy perturbation approach was used by researchers in [17] to solve an epidemic model of EIAV infection. Their findings demonstrate how effective the method is at resolving coupled nonlinear differential equations. This same method has also been applied by [18], where the effect of the disease transmission coefficient on a disease-induced death seizure epidemic model was simulated.

As of date, no research has yet been published that addresses how to restrict the spread of COVID-19 while simultaneously taking therapeutic interventions, immunization, and human cooperation to limit physical interaction into account. The reasons for including these factors in our research are discussed in [8, 19, 20]. Using extracted COVID-19 data in the literature and real-life data obtained from the Nigeria Centre for Disease Control (NCDC) [21], we shall conduct theoretical and numerical analysis on a newly proposed mathematical model of COVID-19 transmission dynamics, which is a modification of the mathematical model in [22], where the dynamics of COVID-19 in relation to isolation class was studied.

## Methods

### Model design

The predicted dynamics of COVID-19 transmission in the population are shown in the schematic diagram below.
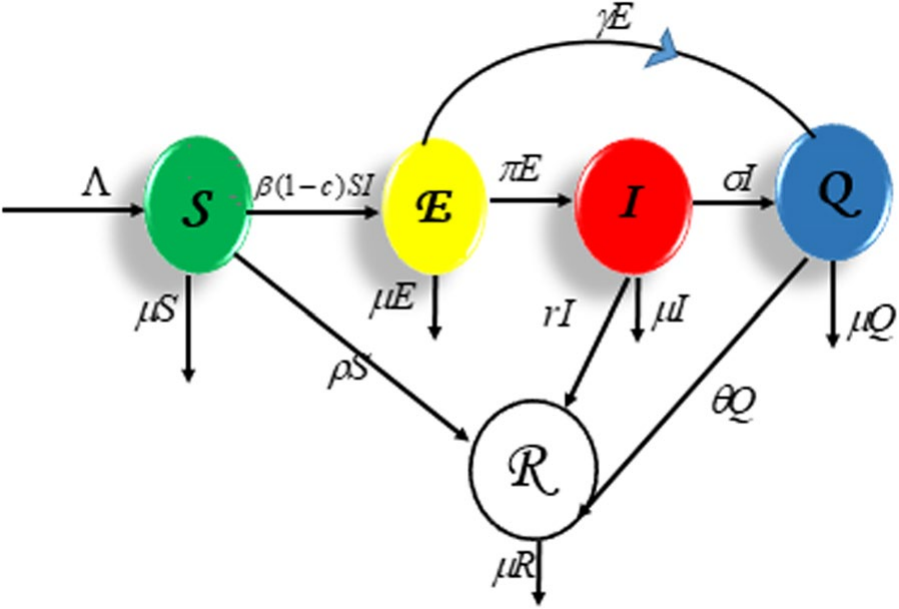


This diagram might be read and expressed mathematically such that

1 $$\left. \begin{aligned} & \frac{{{\text{d}}S(t)}}{{{\text{d}}t}} = \Lambda - \beta (1 - c)S(t)I(t) - (\mu + \rho )S(t), \\ & \frac{{{\text{d}}E(t)}}{{{\text{d}}t}} = \beta (1 - c)S(t)I(t) - (\mu + \gamma + \pi )E(t), \\ & \frac{{{\text{d}}I(t)}}{{{\text{d}}t}} = \pi E(t) - (\sigma + \mu )I(t) - TI(t), \\ & \frac{{{\text{d}}Q(t)}}{{{\text{d}}t}} = \gamma E(t) + \sigma I(t) - (\theta + \mu )Q(t), \\ & \frac{{{\text{d}}R(t)}}{{{\text{d}}t}} = \theta Q(t) - \mu R(t) + \rho S(t) + TI(t). \\ \end{aligned} \right\}$$

### Descriptions

Equation (1) represents a deterministic mathematical model of COVID-19; the population of the model is divided into five classes, namely the susceptible class $S(t)$, exposed class $E(t)$, infected class $I(t)$, quarantine class $Q(t)$ and recovered class $R(t)$. The therapeutic response action T for the sick person I is a piecewise linear function defined in [19]. It varies on the interval $0 \le r < 1$ and is related to the prevalence of infected individuals. Another control parameter under study is “*c*.” It combines curfews, social distancing, and other physical contact restriction measures which are examined on the model to possibly limit the rise of the effective contact rate between the susceptible and infected populations. The immunization of vulnerable persons is represented by parameter $\rho$. We theoretically assume that all susceptible people, including the current and newly recruited members of the class, are administered the vaccine in order to assess its influence on the prevalence of the disease.

For biological reasons, physical contact between the classes of vulnerable and sick individuals should be limited to reduce the disease transmission coefficient. Therefore, it makes sense to create a function $f(\beta ,c)$ in such a way that:

$$f(\beta ,c) = \beta (1 - c).$$

As such

(i) $f(\beta ,0) = \beta$, i.e., without physical restraint, interaction between the classes will continue and the viral coefficient will spread progressively and infection will persist in the system.
(ii) $f(\beta ,1) = 0$, i.e., the model's classes won't interact and disease transmission in the system will stop.
(iii) From (ii), it is valid that $\beta = 0$ when $f(\beta ,1) = 0$. Thus, if there is no infected individual in the system, there is no requirement to prohibit physical contact among the populace.

Since the therapeutic action $T$ on infected individual $I(t)$ I is $rI(t)$, the system of Eq. (1) becomes:

2 $$\begin{aligned} & \frac{{{\text{d}}S(t)}}{{{\text{d}}t}} = \Lambda - \beta (1 - c)S(t)I(t) - (\mu + \rho )S(t), \\ & \frac{{{\text{d}}E(t)}}{{{\text{d}}t}} = \beta (1 - c)S(t)I(t) - (\mu + \gamma + \pi )E(t), \\ & \frac{{{\text{d}}I(t)}}{{{\text{d}}t}} = \pi E(t) - (\sigma + \mu + r)I(t), \\ & \frac{{{\text{d}}Q(t)}}{{{\text{d}}t}} = \gamma E(t) + \sigma I(t) - (\theta + \mu )Q(t), \\ & \frac{{{\text{d}}R(t)}}{{{\text{d}}t}} = \theta Q(t) - \mu R(t) + \rho S(t) + rI(t). \\ \end{aligned}$$

Other factors involved in the dynamics of the disease transmission are described in Tables 1 and 2.

**Table 1:** Description of parameters

Variable	Description
$S(t)$	Time-dependent number of susceptible humans
$E(t)$	Time-dependent number of exposed humans
$I(t)$	Time-dependent number of infected humans
$Q(t)$	Time-dependent number of isolated humans
$R(t)$	Time-dependent number of recovered humans

Incorporated parameters	Description
$c$	Rate at which humans embrace curfew, use of face mask, hand sanitizer and social distancing
$r$	Therapeutic action rate on COVID-19-infected patient
$\rho$	Vaccination rate of susceptible COVID-19 individuals

Parameters	Description
$\Lambda$	Recruitment rate of Individuals
$\beta$	Successful contact rate
$\pi$	Progression rate from exposed to infected
$\sigma$	Progression rate from infected to isolated class
$\gamma$	Progression rate from exposed to isolated class
$\theta$	Recovery rate of isolated individual
$\mu$	Natural death rate

**Table 2:** Values of model’s parameter and references

Parameters	Value	References
$\Lambda$	$750\,\,{\text{day}}^{ - 1}$	[32]
$\beta$	${0}{\text{.0000124}}\,{\text{day}}^{ - 1}$	[33]
$\pi$	${0}{\text{.0000124}}\,{\text{day}}^{ - 1}$	[33]
$\sigma$	$0.010939586\,{\text{day}}^{ - 1}$	[33]
$\gamma$	${4}{\text{.013000000}}\, \times \,{10}^{{ - 8}} \,{\text{day}}^{ - 1}$	[34]
$\theta$	$0.0766169\,{\text{day}}^{ - 1}$	[33]
$\mu$	$0.001466848\,{\text{day}}^{ - 1}$	[33]
$c$	$0$	–
$r$	$0$	–
$\rho$	$0$	–

### Qualitative analysis

#### Existence and uniqueness

A Lipchitz criterion will be employed to ensure that the solution exists and is unique. Thus from Eq. (2), let:

3 $$\begin{aligned} & F_{1} = \Lambda - \left( {\mu + \rho } \right)S - \beta \left( {1 - c} \right)SI, \\ & F_{2} = \left( {1 - c} \right)\beta SI - (\pi + \mu + \gamma )E, \\ & F_{3} = \pi E - \left( {\delta + \mu + r} \right)I,{\kern 1pt} \\ & F_{4} = \gamma E + \delta I - \left( {\mu + \theta } \right)Q, \\ & F_{5} = \theta Q - \mu R + \rho S + rI. \\ \end{aligned}$$

Following the criterion, we obtain the system's partial derivatives.

The partial derivatives of $F_{1} = \Lambda - \left( {\mu + \rho } \right)S - \beta \left( {1 - c} \right)SI$ with respect to the classes yield:

$${\kern 1pt} {\kern 1pt} \left| {\frac{{F_{1} }}{\partial S}} \right| = \left| { - \left( {\mu + \rho } \right)} \right| < \infty ,\left| {{\kern 1pt} {\kern 1pt} \frac{{\partial F_{1} }}{\partial E}} \right| = \left| 0 \right| < \infty ,\left| {{\kern 1pt} \frac{{\partial F_{1} }}{\partial I}} \right| = \left| { - \beta \left( {1 - c} \right)S} \right| < \infty ,\left| {\frac{{\partial F_{1} }}{\partial Q}} \right| = \left| 0 \right| < \infty ,\left| {\frac{{\partial F_{1} }}{\partial R}} \right| = \left| 0 \right| < \infty .$$

Similarly, for $F_{2} = \left( {1 - c} \right)\beta SI - (\pi + \mu + \gamma )E,$ we obtain:

$$\left| {\frac{{\partial F_{2} }}{\partial S}} \right| = \left| {\left( {1 - c} \right)\beta I} \right| < \infty ,\left| {\frac{{\partial F_{2} }}{\partial E}} \right| = \left| {(\pi + \gamma + \mu ){\kern 1pt} {\kern 1pt} } \right| < \infty ,\left| {\frac{{\partial F_{2} }}{\partial I}} \right| = \left| {\left( {1 - c} \right)\beta S} \right| < \infty ,\left| {\frac{{\partial F_{2} }}{\partial Q}} \right| = \left| {0{\kern 1pt} {\kern 1pt} } \right| < \infty ,\left| {\frac{{\partial F_{2} }}{\partial R}} \right| = \left| 0 \right| < \infty .$$

For $$F_{3} = \pi E - \left( {\delta + \mu + r} \right)I{\kern 1pt} {\kern 1pt}$$,

$$\left| {\frac{{\partial F_{3} }}{\partial S}} \right| = \left| 0 \right| < \infty ,\left| {\frac{{\partial F_{3} }}{\partial E}} \right| = \left| \pi \right| < \infty ,\left| {\frac{{\partial F_{3} }}{\partial I}} \right| = \left| { - \left( {\mu + r + \delta } \right)} \right| < \infty ,\left| {\frac{{\partial B_{3} }}{\partial Q}} \right| = \left| 0 \right| < \infty ,\left| {\frac{{\partial B_{3} }}{\partial R}} \right| = \left| 0 \right| < \infty .$$

For $$F_{4} = \gamma E + \delta I - \left( {\mu + \theta } \right)Q$$,

$$\left| {\frac{{\partial F_{4} }}{\partial S}} \right| = \left| 0 \right| < \infty ,\left| {\frac{{\partial F_{4} }}{\partial E}} \right| = \left| \gamma \right| < \infty ,\left| {\frac{{\partial F_{4} }}{\partial I}} \right| = \left| \delta \right| < \infty ,\left| {\frac{{\partial F_{4} }}{\partial Q}} \right| = \left| { - \left( {\mu + \theta } \right)} \right| < \infty ,\left| {\frac{{\partial B_{4} }}{\partial R}} \right| = 0 < \infty .$$

For $$F_{5} = \theta Q - \mu R + \rho S + rI$$,

$$\left| {\frac{{\partial F_{5} }}{\partial S}} \right| = \left| \rho \right| < \infty ,\left| {\frac{{\partial F_{5} }}{\partial E}} \right| = \left| 0 \right| < \infty ,\left| {\frac{{\partial F_{5} }}{\partial I}} \right| = \left| r \right| < \infty ,\left| {\frac{{\partial F_{5} }}{\partial Q}} \right| = \left| \theta \right| < \infty ,\left| {\frac{{\partial F_{5} }}{\partial R}} \right| = \left| { - \mu } \right| < \infty .$$

The partial derivatives of these functions exist and are continuous and bounded; therefore, Eq. (3) exists and has a unique solution in $\Re^{5}$.

#### Nonnegativity of invariant region

The invariant area establishes the viable region for the solution of an epidemiological model, often known as the limit or range for the solution of the model. Any solution outside of this range is neither epidemiologically sound nor relevant to biology. For $t \ge 0$, it is presumed that all of the variables and parameters are positive. As a result, we demonstrate that the area where the model solution of this model lies remains positively invariant for any $t \ge 0$.

Let the total human population be $N(t) = S(t) + E(t) + I(t) + Q(t) + R(t)$. Since the human population varies throughout time,

$$\frac{{{\text{d}}N}}{{{\text{d}}t}} = \frac{{{\text{d}}S}}{{{\text{d}}t}} + \frac{{{\text{d}}E}}{{{\text{d}}t}} + \frac{{{\text{d}}I}}{{{\text{d}}t}} + \frac{{{\text{d}}Q}}{{{\text{d}}t}} + \frac{{{\text{d}}R}}{{{\text{d}}t}},$$

which yields

4 $$\frac{{{\text{d}}N}}{{{\text{d}}t}} = \Lambda - \mu (S + E + I + Q + R).$$

##### Theorem 1

The resulting solutions provided analytically for Eq. (1) are feasible in $\Omega$ for all $t \ge 0$.

##### Proof

Let $D = \left\{ {S,E,I,Q,R} \right\} \in \Re^{5}$ contain the solution of (1) for $\left\{ {S(t),E(t),I(t),Q(t),R(t) \ge 0} \right\}$ and assume that the population is devoid of infection, then *E, I,* and *Q* are set to zero, such that

$$\frac{{{\text{d}}N}}{{{\text{d}}t}} = \Lambda - \mu N$$

and

5 $$\frac{{{\text{d}}N}}{{{\text{d}}t}} \le \Lambda - \mu N.$$

Separating the variables and integrating both sides of Eq. (2) yields

6 $$- \frac{1}{\mu }lin(\Lambda - \mu N) \le t,$$

such that

$$lin(\Lambda - \mu N) \le - \mu t.$$

Thus, solving for the total human population N in (6),

$$\Lambda = \mu N + e^{ - \mu t} .$$

7 $${\text{As}}\,\,t \to \infty \,{\text{we}}\,{\text{have}}\,N \le \frac{\Lambda }{\mu }.$$

This implies that the suggested model in (2) may be investigated in the viable zone.

$$\Omega = \left\{ {\left( {S,E,I,Q,R \in \Re^{5} :N \le \frac{\Lambda }{\mu }} \right)} \right\}$$

#### Nonnegativity of Solution

##### Theorem 2

Given $S > 0,V > 0,E > 0,Q > 0,R > 0$, then the solutions $\left\{ {\left( {S,E,I,Q,R \in \Re^{5} :N \le \frac{\Lambda }{\mu }} \right)} \right\}$ are positive invariant for $t \ge 0$.

##### Proof

From Eq. (1),

$$\frac{{{\text{d}}S(t)}}{{{\text{d}}t}} \ge \left( {\mu + \rho + \beta I - \beta cI} \right){\kern 1pt} {\kern 1pt} {\kern 1pt} {\kern 1pt} S(t).$$

Separating the variables,

$$\frac{{{\text{d}}S(t)}}{S(t)} \ge (\mu + \rho + \beta I - \beta CI){\text{d}}t.$$

Integrating both sides and applying the initial conditions,

$S(t) \ge S_{0} e^{{ - \left( {\mu + \rho + \beta I + \beta CI} \right)t}} \ge 0$. This indicates that $S(t) > 0$ for all $t \ge 0$.

Following the same procedure, we demonstrate the positivity of the other classes.

$$\begin{aligned} & E(t) \ge E_{0} e^{ - (\mu + \pi + \gamma )t} \ge 0. \\ & I(t) \ge I_{0} e^{{ - \left( {\mu + \delta + r} \right)t}} \ge 0. \\ & Q(t) \ge Q_{0} e^{ - (\mu + \theta )t} \ge 0. \\ & R(t) \ge R_{0} e^{ - \mu t} \ge 0. \\ \end{aligned}$$

Thus, $$\left\{ {\left( {S(t),E(t),I(t),Q(t),R(t) > 0,\,\,\forall t \ge 0\,\,in\,\,S(t),E(t),I(t),Q(t),R(t) \in \Re^{5} ;\,and\,N \le \frac{\Lambda }{\mu }\,} \right)} \right\}$$.

The solutions are positive, and this completes the proof.

After satisfying all of the fundamental requirements for an epidemiology model, we conclude that the suggested model is appropriate for studying the dynamics of COVID-19 in the general population.

### Analysis of equilibrium states

#### Disease-free equilibrium (DFE)

By setting the right side of (2) equal to zero, we may determine the equilibrium point devoid of sickness, i.e.,

8 $$\frac{{{\text{d}}S}}{{{\text{d}}t}} = \frac{{{\text{d}}E}}{{{\text{d}}t}} = \frac{{{\text{d}}I}}{{{\text{d}}t}} = \frac{{{\text{d}}Q}}{{{\text{d}}t}} = \frac{{{\text{d}}R}}{{{\text{d}}t}} = 0.$$

We get the following results by solving the equations that emerge from substituting the disease-related classes E, I, and Q to zero:

9 $$(S^{0} ,E^{0} ,I^{0} ,Q,^{0} R^{0} ) = \left( {\frac{\Lambda }{{\left( {\mu + \rho } \right)}},0,0,0,\frac{\Lambda \rho }{{\mu \left( {\mu + \rho } \right)}}} \right).$$

Equation (9) represents the disease-free states of the proposed model.

#### Endemic equilibrium state

Endemic equilibrium, also known as a nonzero equilibrium condition, occurs when a disease persists in a population. Contrary to the disease-free state, $E = I = Q = 0.$

For brevity of terms, we let:

$$f = \beta (1 - c),g = \mu + r,h = \sigma + \mu + r{\kern 1pt} {\kern 1pt} {\kern 1pt} {\kern 1pt} {\kern 1pt} {\kern 1pt} {\kern 1pt} {\kern 1pt} {\kern 1pt} and{\kern 1pt} {\kern 1pt} {\kern 1pt} {\kern 1pt} {\kern 1pt} {\kern 1pt} {\kern 1pt} {\kern 1pt} j = \theta \mu .$$

Thus, the endemic equilibrium state yields

10 $$S^{*} = \frac{{h\left( {\pi + g} \right)}}{f\pi }{\kern 1pt} ,{\kern 1pt}$$

11 $$E^{*} = \frac{\Lambda f\pi - \mu h\pi - \mu hg}{{f\pi \left( {\pi + g} \right)}},$$

12 $$I^{*} = \frac{\Lambda f\pi - \mu h\pi - \mu hg}{{hf\left( {\pi + g} \right)}},$$

13 $$Q^{*} = \frac{{\left( {\Lambda \pi f - \mu \pi h - \mu hg} \right)\left( {\sigma \pi + h\gamma } \right)}}{{hf\left( {\pi + g} \right)}},$$

14 $$R^{*} = \frac{{\left( \begin{gathered} \left( {\rho h^{2} j - \mu h\left( {\theta \sigma + rj} \right) + f\Lambda \left( {\theta \sigma + rj} \right)} \right)\pi^{2} + h\left( {\left( { - \theta \gamma \mu + 2\rho jg} \right)} \right)h \hfill \\ - g\mu \left( {\theta \sigma + rj} \right) + \left. {\theta \gamma \Lambda f} \right)\pi + \left( {gh^{2} \left( {jg\rho - \theta \gamma \mu } \right.} \right. \hfill \\ \end{gathered} \right)}}{{\left( {hf(\pi + g} \right)j\mu \pi }}{\kern 1pt} .$$

#### Basic reproduction number

As defined in [23], the estimated number of secondary cases that can be directly caused by a single case in a population that has reached the peak of an illness is known as the basic reproduction number ${\kern 1pt} {\kern 1pt} R_{0}$.

To compute the basic reproduction number (${\kern 1pt} R_{0}$), we take into account the following two disease-manifested compartments.

15 $$\left. \begin{aligned} & \frac{{{\text{d}}E}}{{{\text{d}}t}} = \beta SI\left( {1 - c} \right) - (\mu + \pi + \gamma )E, \\ & \frac{{{\text{d}}I}}{{{\text{d}}t}} = \pi E - (\mu + r + \sigma )I. \\ \end{aligned} \right\}$$

Let $${\kern 1pt} W_{1} = \beta SI\left( {1 - c} \right) - (\mu + \pi + \gamma )E{\kern 1pt} {\kern 1pt} {\kern 1pt} {\kern 1pt} {\kern 1pt} {\kern 1pt} {\kern 1pt} {\kern 1pt} {\kern 1pt} {\kern 1pt} {\kern 1pt} {\kern 1pt} {\text{and}}\,\,\,\,\,\,\,W_{2} = \pi E - (\mu + r + \sigma )I.$$

${\kern 1pt} {\kern 1pt} R_{0}$ is the spectral radius of the matrix ${\kern 1pt} {\kern 1pt} G = F \times V^{ - 1}$ where ${\kern 1pt} {\kern 1pt} F$ is a matrix constructed with the rate of development of new infection in the model and ${\kern 1pt} {\kern 1pt} V$ comprises the infection's input and outflow in the compartment.

Thus,

16 $$F = \left[ {\begin{array}{*{20}c} {\frac{{\partial W_{1} }}{\partial E}} & {\frac{{\partial W_{1} }}{\partial I}{\kern 1pt} } \\ {\frac{{\partial W{}_{2}}}{\partial E}} & {\frac{{\partial W_{2} }}{\partial I}} \\ \end{array} } \right],$$

such that

17 $$F = \left[ {\begin{array}{*{20}c} {\left( {1 - c} \right)\beta S_{0} } & 0 \\ 0 & 0 \\ \end{array} } \right].$$

Evaluating *F* using disease-free equilibrium (DFE) yields:

18 $$F_{DFE} = \left[ {\begin{array}{*{20}c} {\frac{{\left( {1 - c} \right)\beta \Lambda {\kern 1pt} {\kern 1pt} }}{{\left( {\rho + \mu } \right)}}} & 0 \\ 0 & 0 \\ \end{array} } \right].$$

Constructing $${\kern 1pt} {\kern 1pt} V$$,

$$V^{ + } = \left[ {\begin{array}{*{20}c} 0 & 0 \\ { - \pi } & {\left( {\sigma + \mu + r} \right)} \\ \end{array} } \right]{\kern 1pt} \,\,{\text{and}}\;\;{\kern 1pt} {\kern 1pt} V^{ - } = \left[ {\begin{array}{*{20}c} { - \left( {\mu + \gamma + \pi } \right)} & 0 \\ 0 & 0 \\ \end{array} } \right].$$

Thus,

19 $$V = \left[ {\begin{array}{*{20}c} {\pi + \mu + \gamma } & 0 \\ { - \pi } & {\pi + \mu + r} \\ \end{array} } \right]\,\,{\text{and}}\,\,V^{ - 1} = \left[ {\begin{array}{*{20}c} {\frac{1}{\pi + \mu + \gamma }} & 0 \\ {\frac{\pi }{\pi + \mu + \gamma }} & {\frac{1}{\pi + \mu + r}} \\ \end{array} } \right].$$

Computing $$G = F \times V^{ - 1} ,$$

20 $$\left[ {\begin{array}{*{20}c} {\frac{(1 - c)\beta \Lambda }{{\left( {\pi + \mu + \gamma } \right)(\rho + \mu )}}} & 0 \\ 0 & 0 \\ \end{array} } \right].$$

Computing the eigenvalues, the spectral radius is

21 $$R_{0} = \frac{(1 - c)\beta \Lambda }{{\left( {\pi + \mu + \gamma } \right)(\rho + \mu )}}.$$

Substituting $$S_{0} = \frac{{\Lambda {\kern 1pt} {\kern 1pt} {\kern 1pt} }}{{(\mu + \rho ){\kern 1pt} }}$$,

the basic reproductive ratio is obtained as $R_{0} = \frac{{\pi (1 - c)\beta S_{0} }}{(\mu + \sigma + r)}.$

#### Local stability analysis of disease-free equilibrium

While investigating the stability of the model's equilibria, the following results are demonstrated:

##### Lemma 1

The model's disease-free equilibrium is locally asymptotically stable if ${\kern 1pt} R_{0} < \,{\kern 1pt} 1$ and unstable if ${\kern 1pt} R_{0} > \,{\kern 1pt} 1$.

##### Proof

Consider the Jacobian of Eq. (2), which is given by:

22 $$J(X^{0} ) = \left[ {\begin{array}{*{20}c} { - \left( {\mu + \rho } \right) + (1 - c)\beta I_{0} } & 0 & { - {\kern 1pt} {\kern 1pt} {\kern 1pt} (1 - c)\beta S_{0} } & 0 & 0 \\ {(1 - c)\beta I_{0} } & { - \left( {\mu + \sigma + \gamma } \right)} & {{\kern 1pt} (1 - c)\beta S_{0} } & 0 & 0 \\ 0 & \pi & { - \left( {\mu + \sigma + r} \right)} & 0 & 0 \\ 0 & \gamma & {\sigma {\kern 1pt} } & { - \left( {\mu + \theta } \right)} & 0 \\ 0 & 0 & r & \theta & { - {\kern 1pt} \mu } \\ \end{array} } \right]$$

Substituting $$I_{0} ,S_{0}$$ into $$J(X)$$,

23 $$J(X^{0} ) = \left[ {\begin{array}{*{20}c} { - \left( {\mu + \rho } \right) - \lambda } & 0 & {\frac{{ - {\kern 1pt} {\kern 1pt} {\kern 1pt} (1 - c)\beta }}{{\left( {\mu + \rho } \right)}}} & 0 & 0 \\ 0 & { - \left( {\mu + \sigma + \gamma } \right){\kern 1pt} {\kern 1pt} - {\kern 1pt} \lambda } & {(1 - c)\beta S} & 0 & 0 \\ 0 & \pi & { - \left( {\mu + \sigma + r} \right){\kern 1pt} - \lambda } & 0 & 0 \\ 0 & \gamma & {\sigma {\kern 1pt} } & { - \left( {\mu + \theta } \right){\kern 1pt} - \lambda } & 0 \\ 0 & 0 & r & {\theta {\kern 1pt} {\kern 1pt} } & { - {\kern 1pt} \mu {\kern 1pt} {\kern 1pt} - \lambda } \\ \end{array} } \right]$$

The eigenvalues are then calculated, and the results are as follows:

$$\lambda_{1} = - \left( {\mu + \rho } \right),\,\,\lambda_{2} = - \left( {\pi + \mu + \gamma } \right),\,\,\,\lambda_{3} = 0{\kern 1pt} - \left( {\pi + \sigma + r} \right){\kern 1pt} ,{\kern 1pt} {\kern 1pt} {\kern 1pt} {\kern 1pt} {\kern 1pt} {\kern 1pt} {\kern 1pt} {\kern 1pt} {\kern 1pt} \lambda_{4} = - \left( {\mu + \theta } \right) - \mu ,\,\,\,\lambda_{5} = - \mu {\kern 1pt} .{\kern 1pt} {\kern 1pt}$$

We infer that the disease-free equilibrium exists since the eigenvalues are all negative.

#### Global stability at disease-free equilibrium

##### Lemma 2

Applying the Lyapunov function approach to the global stability of the disease-free equilibrium state of the model,

$$V(S,E,I,Q,R) = C_{1} E + C_{2} I,$$

$$\begin{aligned} & \frac{dV}{{dt}} = C_{1} \mathop E\limits^{ \bullet } + C_{2} \mathop I\limits^{ \bullet } , \\ & \frac{dV}{{dt}} = C_{1} \left( {\left( {1 - c} \right)\beta SI - \left( {\pi + \mu + \gamma } \right)E} \right) + C_{2} \left( {\pi E - \left( {\sigma + \mu + r} \right)I} \right){\kern 1pt} , \\ & \frac{dV}{{dt}} = \left( {C_{2} \pi - C_{1} \left( {\pi + \mu + \gamma } \right)} \right)E + \left( {C_{1} \left( {\left( {1 - c} \right)\beta S - \left( {\sigma + \mu + r} \right)C_{2} } \right)} \right)I \le C_{2} \pi E - C_{1} \left( {\pi + \mu + \gamma } \right) + C_{1} \left( {1 - c} \right)\beta SI \\ & - C_{2} \left( {\pi E - \left( {\sigma + \mu + r} \right)I} \right) \le C_{2} \pi E - C_{1} \left( {\pi + \mu + \gamma } \right)E + \frac{{C_{1} \left( {1 - c} \right)\pi \beta I}}{{\left( {\rho + \mu } \right)}} - \left( {\sigma + \mu + r} \right)C_{2} I, \\ \end{aligned}$$

Suppose

$${\kern 1pt} C_{1} = \frac{1}{{\left( {\pi + \mu + \gamma } \right)}}{\kern 1pt} \,{\kern 1pt} {\text{and}}\,{\kern 1pt} C_{2} = \frac{{\pi \beta \left( {1 - c} \right)}}{{\left( {\pi + \mu + \gamma } \right)\left( {\rho + \mu } \right)\left( {\sigma + \mu + r} \right)}}.$$

Since $$R{\kern 1pt}_{0} = \frac{{\pi (1 - c)\beta \Lambda {\kern 1pt} {\kern 1pt} {\kern 1pt} }}{(\mu + \sigma + r)(\mu + \rho )},$$

Then we have $R{\kern 1pt}_{0} I - I = \left( {R{\kern 1pt}_{0} - 1} \right)I.$

Therefore, $$V^{^{\prime}} \le \left( {\pi + \mu + \gamma } \right)\left( {R{\kern 1pt}_{0} - 1} \right)I{\kern 1pt} {\kern 1pt} {\kern 1pt} {\kern 1pt}$$.

It is critical to note that $V^{^{\prime}} = 0$ only when $E = 0$. Substituting $E = 0$ in the model equation yields $\frac{\Lambda }{{\left( {\mu + \rho } \right)}}$. According to LaSalle's invariance principle, the disease-free equilibrium E_0_ is globally asymptotically stable if $R_{0} < 1$.

### Sensitivity Analysis

We used the sensitivity index analysis to assess the resilience of the model parameter based on the fundamental reproduction number. The sensitivity indices of each parameter can be computed with the following formula:

24 $$\chi_{{R_{0} }}^{\beta } = \frac{{\partial R_{0} }}{\partial \beta } \times \frac{\beta }{{R_{0} }}{\kern 1pt} .$$

According to the findings, the three control variables yield low sensitivity indices. They are negative, but given how close to unity they are, it is hard to underestimate their effectiveness in lowering the basic reproduction number (Table 3).

**Table 3:** Sensitivity indices of $R_{0} = {0}{\text{.01409544898}}$ to model’s parameters

Parameter	Flexibility index
$\pi$	1
$\Lambda$	1
$\beta$	1
c	− 1
$\rho$	− 0.99
$\mu$	$- {7}.{52} \times 10^{ - 7}$
$\sigma$	− 0.03105469684
r	− 0.99689452992

### Homotopy perturbation method

One of the aims of this research is to do numerical simulations on the mathematical model. To do so, we must first establish an approximation solution to the mathematical model. In pursuit of this, we explore the methods of applying the homotopy perturbation. The method's analysis is explained more below. Hence, consider the following differential equation:

25 $$\Delta (\omega ) = k(r),\quad r \in \Phi .$$

Subject to the boundary condition

26 $$\Psi (\omega ,\omega_{n} ) = 0\quad r \in \Pi .$$

Operator $\Delta$ denotes the differential operator, the boundary operator is $\Psi$, k(r) is an analytic function, the boundary of the domain $\Phi$ is denoted by $\Pi$, and $\omega_{n}$ is the normal vector derivative drawn externally from $\Phi$. We can split the operator $\Delta (\omega )$ into two parts such that

27 $$\Delta (\omega ) = L_{T} (\omega ) + N_{T} (\omega ).$$

The operator $L_{T} (\omega ),\,N_{T} (\omega )$ denotes the linear and nonlinear term, respectively, such that Eq. (25) implies

28 $$L_{T} (\omega ) + N_{T} (\omega ) = k(r),\quad r \in \Phi .$$

We can construct a homotopy for (28) so that

29 $$H(f,p) = (1 - p)\left[ {L_{T} (f) - L_{T} (\omega_{0} )} \right] + p\left[ {\Delta (f) - k(r)} \right] = 0,$$

where *p* is an embedding parameter which can undergo a deformation process of changing from [0, 1]. Equation (29) is further simplified to obtain:

30 $$H(f,p) = L_{T} (f) - L_{T} (\omega_{0} ) + p[L_{T} (\omega_{0} )] + p\left[ {N_{T} (\omega_{0} ) - k(r)} \right] = 0,$$

as $p \to 0,$ Eq. (30) gives:

31 $$H(f,0) = L_{T} (f) - L_{T} (\omega_{0} ) = 0.$$

And when $$p \to 1,$$

32 $$H(f,1) = \Delta (f) - k(r) = 0.$$

We can naturally assume the solution (28) as a power series such that

33 $$f(t) = f_{0} (t) + pf_{1} (t) + p^{2} f_{2} (t) + \cdots p^{n} f_{n} (t).$$

Such that evaluating (30) using (33), and comparing coefficients of equal powers of p.

The values of $f_{0} (t),f_{1} (t),\,f_{2} (t)$ are obtained by solving the resulting ordinary differential equations. Thus, the approximate solution of (25) is:

34 $$f(t) = \mathop {\lim }\limits_{p \to 1} f_{n} (t) = f_{0} (t) + f_{1} (t) + f_{2} (t) + \cdots$$

### Numerical simulation

In this part, we use the homotopy perturbation approach to conduct the numerical simulation that produces the SEIQR epidemic model's approximate solution. Constructing a homotopy for (1),

35 $$\begin{aligned} & (1 - p)\frac{{{\text{d}}S(t)}}{{{\text{d}}t}} + p\left( {\frac{{{\text{d}}S(t)}}{{{\text{d}}t}} + \Lambda - \beta (1 - c)S(t)I(t) - (\mu + \rho )S(t)} \right) = 0, \\ & (1 - p)\frac{{{\text{d}}E(t)}}{{{\text{d}}t}} + p\left( {\frac{{{\text{d}}E(t)}}{{{\text{d}}t}} + \beta (1 - c)S(t)I(t) - (\mu + \gamma + \pi )E(t)} \right) = 0, \\ & (1 - p)\frac{{{\text{d}}I(t)}}{{{\text{d}}t}} + p\left( {\frac{{{\text{d}}I(t)}}{{{\text{d}}t}} + \pi E(t) - (\sigma + \mu + r)I(t)} \right) = 0, \\ & (1 - p)\frac{{{\text{d}}Q(t)}}{{{\text{d}}t}} + p\left( {\frac{{{\text{d}}Q(t)}}{{{\text{d}}t}} + \gamma E(t) + \sigma I(t) - (\theta + \mu )Q(t)} \right) = 0, \\ & (1 - p)\frac{{{\text{d}}R(t)}}{{{\text{d}}t}} + p\left( {\frac{{{\text{d}}R(t)}}{{{\text{d}}t}}\theta Q(t) - \mu R(t) + \rho S(t) + rI(t)} \right) = 0. \\ \end{aligned}$$

The approximate solution of (1) can be assumed as:

36 $$\begin{aligned} & S(t) = s_{0} (t) + ps_{1} (t) + p^{2} s_{2} (t) + \ldots p^{n} s_{n} (t) \\ & E(t) = e_{0} (t) + pe_{1} (t) + p^{2} e_{2} (t) + \ldots p^{n} s_{n} (t) \\ & I(t) = i_{0} (t) + pi_{1} (t) + p^{2} i_{2} (t) + \ldots p^{n} s_{n} (t) \\ & Q(t) = q_{0} (t) + pq_{1} (t) + p^{2} q_{2} (t) + \ldots p^{n} s_{n} (t) \\ & R(t) = r_{0} (t) + pr_{1} (t) + p^{2} r_{2} (t) + \ldots p^{n} s_{n} (t) \\ \end{aligned}$$

Substituting (36) into (35) and comparing coefficients of equal powers of $p$,

37 $$p^{0} :\quad \mathop {i_{0} }\limits^{ \bullet } (t) = 0,\,\,\,\,\mathop {e_{0} }\limits^{ \bullet } (t) = 0,\,\,\,\,\mathop {i_{0} }\limits^{ \bullet } (t) = 0\,,\,\,\mathop {q_{0} }\limits^{ \bullet } (t) = 0,\,\,\,\,\mathop {r_{0} }\limits^{ \bullet } (t) = 0.\,$$

Solving (37) yields

38 $$s_{0} (t) = s_{0} ,\,\,\,\,e_{0} (t) = e_{0} ,\,\,\,i_{0} (t) = i_{0} ,\,\,q_{0} (t) = q_{0} ,\,\,r_{0} (t) = r_{0}$$

Similarly, comparing the coefficients of $p^{1}$,

39 $$\begin{aligned} & \frac{{{\text{d}}S_{1} (t)}}{{{\text{d}}t}} = \Lambda - \beta (1 - c)S_{0} (t)I_{0} (t) - (\mu + \rho )S_{0} (t), \\ & \frac{{{\text{d}}E_{1} (t)}}{{{\text{d}}t}} = \beta (1 - c)S_{0} (t)I_{0} (t) - (\mu + \gamma + \pi )E_{0} (t), \\ & \frac{{{\text{d}}I_{1} (t)}}{{{\text{d}}t}} = \pi E_{0} (t) - (\sigma + \mu + r)I_{0} (t), \\ & \frac{{{\text{d}}Q_{1} (t)}}{{{\text{d}}t}} = \gamma E_{0} (t) + \sigma I_{0} (t) - (\theta + \mu )Q_{1} (t), \\ & \frac{{{\text{d}}R_{1} (t)}}{{{\text{d}}t}} = \theta Q_{0} (t) - \mu R_{0} (t) + \rho S_{0} (t) + rI_{0} (t). \\ \end{aligned}$$

Evaluating (39) using (38), and then solving the resulting system of equations, produces

40 $$\begin{aligned} & S_{1} (t) = \left( {\Lambda - \beta (1 - c)s_{0} i_{0} - (\mu + \rho )s_{0} } \right)t. \\ & E_{1} (t) = \left( {\beta (1 - c)s_{0} i_{0} - (\mu + \gamma + \pi )e_{0} } \right)t. \\ & I_{1} (t) = \left( {\pi e_{0} - (\sigma + \mu + r)i_{0} } \right)t. \\ & Q_{1} (t) = \left( {\gamma e_{0} + \sigma i_{0} - (\theta + \mu )q} \right)t. \\ & R_{1} (t) = \left( {\theta q_{0} - \mu r_{0} + \rho s_{0} + ri_{0} } \right)t. \\ \end{aligned}$$

The coefficients of $p^{2}$ equally yield:

$$\begin{aligned} & \frac{{{\text{d}}S_{2} (t)}}{{{\text{d}}t}} = \Lambda - \beta (1 - c)S_{1} (t)I_{1} (t) - (\mu + \rho )S_{1} (t), \\ & \frac{{{\text{d}}E_{2} (t)}}{{{\text{d}}t}} = \beta (1 - c)S_{1} (t)I_{1} (t) - (\mu + \gamma + \pi )E_{1} (t), \\ & \frac{{{\text{d}}I_{2} (t)}}{{{\text{d}}t}} = \pi E_{1} (t) - (\sigma + \mu + r)I_{1} (t), \\ & \frac{{{\text{d}}Q_{2} (t)}}{{{\text{d}}t}} = \gamma E_{1} (t) + \sigma I_{1} (t) - (\theta + \mu )Q_{1} (t), \\ & \frac{{{\text{d}}R_{2} (t)}}{{{\text{d}}t}} = \theta Q_{1} (t) - \mu R_{1} (t) + \rho S_{1} (t) + rI_{1} (t). \\ \end{aligned}$$

The second approximations are obtained by solving these equations.

$$\begin{aligned} & S_{2} (t) = \frac{{t^{2} }}{2}\left( \begin{gathered} - 3\beta ci_{0} \mu s_{0} - 2\beta ci_{0} \rho s_{0} + \beta cs_{0} \pi e_{0} + \beta cs_{0} i_{0} \sigma - \beta cs_{0} ri_{0} - \mu \Lambda \hfill \\ + e_{0} \mu^{2} - \Lambda \rho + \rho^{2} s_{0} + 3\mu i_{0} s_{0} + 2\beta i_{0} \rho s_{0} - 2\beta^{2} i_{0}^{2} s_{0} + \beta ci_{0} \Lambda \hfill \\ + \beta^{2} c^{2} i_{0}^{2} s_{0} - \beta s_{0} \pi e_{0} + \beta s_{0} \sigma i_{0} + \beta s_{0} ri_{0} - \beta i_{0} \Lambda + \beta^{2} i_{0}^{2} s_{0} + 2\mu \rho s_{0} . \hfill \\ \end{gathered} \right) \\ & E_{2} (t) = - \frac{{t^{2} }}{2}\left( \begin{gathered} \mu^{2} e_{0} + \pi^{2} e_{0} + \gamma^{2} e_{0} + 2\mu e_{0} \pi + 2e_{0} \gamma \pi + 2e_{0} \gamma \mu - 3\beta i_{0} \mu s_{0} - \beta i\rho s_{0} \hfill \\ + 2\beta^{2} i_{0}^{2} cs_{0} - \beta ci_{0} \Lambda + \beta^{2} c^{2} i_{0}^{2} s_{0} - \beta s_{0} ri_{0} + \beta S_{0} \sigma i_{0} - \beta s_{0} ri_{0} + \beta s_{0}^{2} \pi e_{0}^{2} - \beta \pi s_{0} i_{0} \hfill \\ + \gamma \beta s_{0} i_{0} + \beta \pi cs_{0} i_{0} + \gamma \beta cs_{0} i_{0} + 3\beta cs_{0} \mu i_{0} + \beta cs_{0} \rho i_{0} + \beta cs_{0} \sigma i_{0} + \beta cs_{0} ri_{0} - \beta cs_{0} \pi e_{0} \hfill \\ + \beta i_{0} \Lambda - \beta^{2} i_{0}^{2} s_{0} . \hfill \\ \end{gathered} \right) \\ & I_{2} (t) = + \frac{{t^{2} }}{2}\left( \begin{gathered} e_{0} \pi^{2} - \pi \beta s_{0} i_{0} + 2e_{0} \mu \pi + e_{0} \pi \gamma + \pi \beta cs_{0} i_{0} + \sigma \pi e_{0} - i_{0} \sigma^{2} - 2i_{0} r\sigma - 2i_{0} \mu \sigma \hfill \\ - 2i_{0} \mu r - i_{0} \mu^{2} + r\pi e_{0} - i_{0} r^{2} . \hfill \\ \end{gathered} \right) \\ & q_{2} (t) = + \frac{{t^{2} }}{2}\left( \begin{gathered} - \sigma^{2} i_{0} + \sigma \pi e_{0} - 2\sigma \mu i_{0} + \beta Ns_{0} \gamma e_{0} - \gamma^{2} e_{0} + \gamma \beta Ns_{0} i_{0} \hfill \\ - \pi \gamma e_{0} - 2\mu \gamma e_{0} + \mu^{2} q_{0} + 2q_{0} \theta \mu - \theta \sigma i_{0} - \theta \gamma e_{0} + \theta^{2} q_{0} . \hfill \\ \end{gathered} \right) \\ & r_{2} (t) = \frac{{t^{2} }}{2}\left( \begin{gathered} - 2\mu \rho s_{0} + r_{0} \mu^{2} - 2i_{0} r - 2q_{0} \theta \mu + \theta \gamma e_{0} - q_{0} \theta^{2} + i_{0} \theta \sigma \hfill \\ + \Lambda \rho - \rho^{2} s_{0} + \beta ci_{0} s_{0} \rho - \beta i_{0} s_{0} \rho + r\pi e_{0} - i_{0} r\sigma - i_{0} r^{2} . \hfill \\ \end{gathered} \right) \\ \end{aligned}$$

This continues till the desired number of iteration is computed. For the purpose of this research, we performed three iterations. Due to cumbersome number of terms, we present the iteration code instead of the computed results.

- **>ic** := {s[2](t), e[0](t) = e[0], e[1](t) = (-Pi*e[0]+beta*s[0]*i[0]-e[0]*mu-e[0]*gamma-beta*c*s[0]*i[0])*t, e[2](t) = (1/2)*t^2*(-beta^2*c^2*i[0]^2*s[0]-3*beta*i[0]*mu*s[0]-beta*i[0]*rho*s[0]+2*beta^2*i[0]^2*c*s[0]-beta*c*i[0]*Lambda-beta*s[0]*Pi*i[0]-gamma*beta*s[0]*i[0]-beta*s[0]*sigma*i[0]+e[0]*gamma^2+e[0]*Pi^2+beta*s[0]*Pi*e[0]+2*e[0]*mu*Pi-beta^2*i[0]^2*s[0]+e[0]*mu^2-beta*s[0]*r*i[0]+beta*i[0]*Lambda+2*e[0]*gamma*mu+2*e[0]*gamma*Pi-Pi*beta*c*s[0]*e[0]+beta*c*s[0]*sigma*i[0]+Pi*beta*c*s[0]*i[0]+gamma*beta*c*s[0]*i[0]+3*beta*c*i[0]*mu*s[0]+beta*c*i[0]*rho*s[0]+beta*c*s[0]*r*i[0]), i[0](t) = i[0], i[1](t) = (Pi*e[0]-sigma*i[0]-i[0]*r-i[0]*mu)*t, i[2](t) = -(1/2)*t^2*(e[0]*Pi^2-beta*s[0]*Pi*i[0]+2*e[0]*mu*Pi+e[0]*gamma*Pi+Pi*beta*c*s[0]*i[0]+sigma*Pi*e[0]-i[0]*sigma^2-2*i[0]*r*sigma-2*i[0]*mu*sigma-2*i[0]*r*mu-i[0]*mu^2+r*Pi*e[0]-i[0]*r^2), q[0](t) = q[0], q[1](t) = (e[0]*gamma-q[0]*theta-mu*q[0]+sigma*i[0])*t, q[2](t) = -(1/2)*t^2*(2*e[0]*gamma*mu-2*q[0]*theta*mu-mu^2*q[0]+2*i[0]*mu*sigma+e[0]*gamma*Pi-gamma*beta*s[0]*i[0]+e[0]*gamma^2+gamma*beta*c*s[0]*i[0]-sigma*Pi*e[0]+i[0]*sigma^2+i[0]*r*sigma+theta*gamma*e[0]-q[0]*theta^2+theta*sigma*i[0]), r[0](t) = r[0], r[1](t) = (rho*s[0]-mu*r[0]+i[0]*r+q[0]*theta)*t, r[2](t) = (1/2)*t^2*(-2*mu*rho*s[0]+r[0]*mu^2-2*i[0]*r*mu-2*q[0]*theta*mu+theta*gamma*e[0]-q[0]*theta^2+theta*sigma*i[0]+Lambda*rho-rho^2*s[0]+beta*c*i[0]*rho*s[0]-beta*i[0]*rho*s[0]+r*Pi*e[0]-i[0]*r*sigma-i[0]*r^2), s[0](t) = s[0], s[1](t) = (Lambda-mu*s[0]-rho*s[0]+beta*c*s[0]*i[0]-beta*s[0]*i[0])*t}
- **>S3**: =dsolve({eval(diff(s[3](t), t)-mu*s[2](t)+beta*s[0](t)*i[2](t)-beta*c*s[1](t)*i[1](t)+rho*s[2](t)-beta*c*s[0](t)*i[2](t) beta*c*s[2](t)*i[0](t)+beta*s[2](t)*i[0](t)+beta*s[1](t)*i[1](t), ic), s[3](0) = 0});
- **>E3**: =dsolve({eval(diff(e[3](t), t)-beta*s[1](t)*i[1](t)+beta*c*s[1](t)*i[1](t)+beta*c*s[2](t)*i[0](t)-beta*s[2](t)*i[0](t)+gamma*e[2](t)+beta*c*s[0](t)*i[2](t)-beta*s[0](t)*i[2](t)+mu*e[2](t)+Pi*e[2](t), ic), e[3](0) = 0});
- **> Iota3** := dsolve({eval(diff(i[3](t), t)+mu*i[2](t)-Pi*e[2](t)+sigma*i[2](t)+ +r*i[2](t), ic), i[3](0) = 0});
- **> Q3 :=** dsolve({eval(diff(q[3](t), t)+mu*q[2](t)+theta*q[2](t)-sigma*i[2](t)-gamma*e[2](t), ic), q[3](0) = 0});
- **>** **R3:=** dsolve({eval(-rho*s[2](t)+mu*r[2](t)-r*i[2](t)+diff(r[3](t), t)-theta*q[2](t), ic), r[3](0) = 0});

## Results

The solution for each class is obtained by taking the sum of its obtained approximations such that: $S(t) = \sum\limits_{n = 0}^{3} {s_{n} (t)} ,\,\,\,E(t) = \sum\limits_{n = 0}^{3} {e_{n} (t),\,\,I(t) = \sum\limits_{n = 0}^{3} {i_{n} (t)} ,\,\,Q(t) = \sum\limits_{n = 0}^{3} {q_{n} (t)} \,,\,R(t) = \sum\limits_{n = 0}^{3} {r_{n} (t)} }$.

Additionally, the outcome is assessed to project the dynamics of the disease using actual data from the Nigeria Center for Disease Control (NCDC) report from December 1, 2020. $E(0) = 2003,\,$ $\,I(0) = 416,\,$ $\,Q(0) = 404,\,$ $\,R(0) = 115$ as initial conditions. The validity of the mathematical model is established by testing it on a real-life population of Ikeja, Lagos Nigeria, defined as $S(0) = 470200$ [24]:

$$S(t): = {470200 + }\left( \begin{gathered} { - 3238}{\text{.700958}} \hfill \\ { - 470200}\rho \hfill \\ { + 2425}{\text{.479680c}} \hfill \\ \end{gathered} \right){\text{t + }}\left( \begin{gathered} {38}{\text{.05527610}} \hfill \\ + {7227}{\text{.401916}}\rho \hfill \\ + {470200}\rho^{{2}} \hfill \\ { - 47}{\text{.86324479c}} \hfill \\ { - 4850}{\text{.959360c}}\rho \hfill \\ { - 2425}{\text{.479680cr}} \hfill \\ { + 12}{\text{.51159438}}c^{{2}} \hfill \\ { + 2425}{\text{.479680r}} \hfill \\ \end{gathered} \right)\frac{{{\text{t}}^{{2}} }}{2}{ - }\left( \begin{gathered} {75}{\text{.28685478r - 7276}}{\text{.439040c}}\rho {\text{r}} \hfill \\ { - 112}{\text{.8216379cr - 7276}}{.439040}\rho^{{2}} {\text{c}} \hfill \\ { - 2425}{\text{.479680c}}r^{{2}} { + 37}{\text{.53478314}}c^{{2}} {\text{r}} \hfill \\ { + 7276}{\text{.439040}}\rho {\text{r - 147}}{\text{.4585344c}}\rho \hfill \\ { - 1}{\text{.232160531c + 120}}{.5280693}\rho \hfill \\ { + }{\text{.6791905779 + 11216}}{.10287}\rho^{{2}} \hfill \\ { - 0}{\text{.06453980844}}c^{{3}} { + 37}{\text{.53478314}}\rho c^{{2}} \hfill \\ { + 2425}{\text{.479680}}r^{{2}} { + }{\text{.6264982021}}c^{{2}} \hfill \\ { + 470200}\rho^{{3}} \hfill \\ \end{gathered} \right)\frac{{{\text{t}}^{{3}} }}{6} + \cdots$$

$$E(t): = {2003 + }\left( \begin{gathered} {2418}{\text{.797179}} \hfill \\ { - 2425}{\text{.479680c}} \hfill \\ \end{gathered} \right){\text{t + }}\left( \begin{gathered} { - 2425}{\text{.479680}} \hfill \\ + {2425}{\text{.479680cr}} \hfill \\ + {2425}{\text{.479680c}}\rho^{{2}} \hfill \\ + {47}{\text{.89152133c}} \hfill \\ { - 2425}{\text{.479680}}\rho \hfill \\ { - 35}{\text{.35763249}} \hfill \\ - {12}{\text{.51159438}}c^{{2}} \hfill \\ \end{gathered} \right)\frac{{{\text{t}}^{{2}} }}{2}{ - }\left( \begin{gathered} { - 75}{\text{.31513133r}} + {4850}{\text{.959360c}}\rho {\text{r}} \hfill \\ + {112}{\text{.8499145cr}} + {2425}{\text{.479680}}\rho^{{2}} {\text{c}} \hfill \\ - {37}{\text{.53478314c}}r^{{2}} { - 4850}{\text{.959360}}\rho {\text{r}} \hfill \\ { - 0}{\text{.6706342282 - 91}}{\text{.55984549c}}\rho \hfill \\ { + 1}{\text{.232812862}}c{ - 66}{\text{.53665672}}\rho \hfill \\ { + 2425}{\text{.479680}}\rho^{{2}} + {0}{\text{.06453980844}}c^{{3}} \hfill \\ { - 25}{\text{.02318876}}\rho c^{{2}} { - 2425}{\text{.479680}}r^{{2}} \hfill \\ { - 0}{\text{.6266440639}}c^{{2}} \hfill \\ \end{gathered} \right)\frac{{{\text{t}}^{{3}} }}{6} + \cdots$$

$$I(t): = {416 + }\left( \begin{gathered} { - 1}{\text{.814844532}} \hfill \\ {\text{ - 416r}} \hfill \\ \end{gathered} \right){\text{t - }}\left( \begin{gathered} { - 0}{\text{.03612056076}} \hfill \\ + {0}{\text{.02817922292c}} \hfill \\ + {3}{\text{.652959917r}} \hfill \\ - 416r^{{2}} \hfill \\ \end{gathered} \right)\frac{{{\text{t}}^{{2}} }}{2} + \left( \begin{gathered} { - 0}{\text{.08044055730r}} + {0}{\text{.05635844584cr}} \hfill \\ - {0}{\text{.0005703853552}} - {416}r^{{3}} + {0}{\text{.02817922292c}}\rho \hfill \\ { - 0}{\text{.0006809149044c - 0}}{.02817922292}\rho \hfill \\ { - 5}{\text{.491075303}}r^{2} { - 0}{\text{.0001453597035}}c^{{2}} \hfill \\ \end{gathered} \right)\frac{{{\text{t}}^{{3}} }}{6} + \cdots$$

$$Q(t): = {404 + }\left( {{ - 31}{\text{.84119399}}} \right){\text{t - }}\left( \begin{gathered} { - 2}{\text{.543544130}} \hfill \\ + {0}{\text{.00009733449956c}} \hfill \\ + {\text{.4550867776r}} \hfill \\ \end{gathered} \right)\frac{{{\text{t}}^{{2}} }}{2} + \left( \begin{gathered} {0}{\text{.04027916659r}} + {0}{\text{.00009733449956cr}} \hfill \\ + {0}{\text{.00009733449956c}}\rho { - 0}{\text{.00002112395177c}} \hfill \\ { - 0}{\text{.2032966070 - 0}}{.00009733449956}\rho \hfill \\ { - }{\text{.455086777}}r^{{2}} - {5}{\text{.020902824x10}}^{{ - 7}} c^{2} \hfill \\ \end{gathered} \right)\frac{{{\text{t}}^{{3}} }}{6} + \cdots$$

$$R(t): = {115 + }\left( \begin{gathered} {470200}\rho + {\text{416r}} \hfill \\ { + 30}{\text{.57089998}} \hfill \\ \end{gathered} \right){\text{t - }}\left( \begin{gathered} { - 4801}{\text{.922236}}\rho \hfill \\ { - 2}{\text{.541209224}} \hfill \\ { - 3}{\text{.197873140r}} \hfill \\ {470200}\rho^{{2}} { - 416}r^{{2}} \hfill \\ { + 2425}{\text{.479680}}c\rho \hfill \\ \end{gathered} \right)\frac{{{\text{t}}^{{2}} }}{2} - \left( \begin{gathered} { - 0}{\text{.01188483329r}} + {2425}{\text{.479680c}}\rho {\text{r}} \hfill \\ + {0}{\text{.02817922292cr}} + {4850}{\text{.95936}}\rho^{{2}} c \hfill \\ + {55}{\text{.92696542c}}\rho + {0}{\text{.000007457467618c}} \hfill \\ { - 54}{\text{.01968915}}\rho + {8790}{\text{.623194}}\rho^{{2}} \hfill \\ - {12}{\text{.51159438}}\rho c^{{2}} - {5}{\text{.035988525}}r^{{2}} \hfill \\ - {0}{\text{.2033269399 - 470200}}\rho^{{3}} {\text{ - 416r}}^{{3}} \hfill \\ + {2425}{\text{.479680}}\rho {\text{r}} \hfill \\ \end{gathered} \right)\frac{{{\text{t}}^{{3}} }}{6} + \cdots$$

### Convergence of HPM

The convergence of the homotopy perturbation approach strictly depends on the contraction of the approximation solution to the exact solution [25].

#### Theorem 3

Let there exist a mapping $k:m \to n$ defined on two Banach spaces $m$, $n$ for all $s,t \in m$ then $\left\| {k(s) - k(t)} \right\|_{n} \le \delta \left\| {s - t} \right\|_{m}$, $0 < \delta < 1$ such that the sequence $s_{r + 1} = k^{n} (s_{0} ) = \tau (s_{0} )$ for some $s_{0} \in m$ which converges to a unique fixed point $k$ [25].

#### Proof

We consider a Picard sequence $s_{r + 1} = k(s_{r} ) \subseteq n$ to prove the theorem. It is required to show that $s_{r}$ is convergent in $n$ for all $r \ge \nu$ such that $\left\| {s_{r} - s_{v} } \right\| \le \left\| {s_{r} - s_{r + 1} } \right\| + \left\| {s_{r + 1} - s_{r + 2} } \right\| + + \left\| {s_{r + 2} - s_{r + 3} } \right\| + \cdots$ $+ \left\| {s_{r - 1} - s_{v} } \right\|$. **T**he proof is defined by applying mathematical induction on the contractive property of contraction c such that $\left\| {s_{r} - s_{r + 1} } \right\| \le \delta^{r} \left\| {s_{0} - s_{1} } \right\|$. This implies $\mathop {\lim }\limits_{v \to \infty } \left\| {s_{r} - s_{v} } \right\| \le \frac{{\delta^{r} }}{1 + \delta }\left\| {s_{0} - s_{1} } \right\| = 0\,\,as\,\,r \to \infty$.

This proves that $(s_{r} )$ is convergent in $n$ and through completeness of $n$, we can find $\omega \in n$: $\mathop {\lim }\limits_{r \to \infty } (s_{r} ) = \omega \in n$. Clearly, contraction C ensures the continuity of $k$. Thus, $\omega = \mathop {\lim }\limits_{r \to \infty } s_{r + 1} = s_{v}$.

#### Lemma 3

The proposed mathematical model's convergence of $s_{r}$ to $s_{v}$ cannot be asserted since no exact solution to the model exists.

#### Proof

The rate of convergence of $s_{r}$ depends on the following conditions as suggested by He [16, 26]:

(i) The nth-order derivative of $f(t)$ with respect to t in (33) must be small as parameter $p$ may be relatively large as $p \to 1$
(ii) ii. $\left\| {\ell^{ - 1} {{\partial f(t)} \mathord{\left/ {\vphantom {{\partial f(t)} {\partial v}}} \right. \kern-0pt} {\partial v}}} \right\| < 1$ so that the series converge.

This rate is examined in the following section.

### Error analysis

In numerical computations of series solutions, error analysis is crucial. In this section, we utilize the convergence requirements from Lemma 1 to perform an error analysis on the approximate results of each compartment of the model using Maclaurin's estimation of maximum truncation error at predefined baseline values of the parameters given in Table 2.

#### Definition

The truncation error of an $n{\text{th}}$-order Maclaurin’s polynomial of a function $f(t)$ with $n + 1$ derivatives on the interval $\left| t \right| \le r$ and in the interval $\left| {f^{n + 1} (t)} \right| \le M$ has the following bound as maximum error $E_{n} (t) = \mathop {\max }\limits_{t \in [0,r]} \left[ {\frac{M}{(n + 1)!} \cdot \left| t \right|^{{^{n + 1} }} } \right]$.

To compute the third-order maximum truncation error of the model's class series results on the interval $[0,1]$, we construct the following functions:

$$\begin{aligned} & S_{e3} (t) = \mathop {\max }\limits_{t \in [0,1]} \left[ {\frac{{d^{3} S(t)}}{{dt^{3} }} \cdot \frac{{\left| t \right|^{3} }}{3!}} \right],E_{e3} (t) = \mathop {\max }\limits_{t \in [0,1]} \left[ {\frac{{d^{3} E(t)}}{{dt^{3} }} \cdot \frac{{\left| t \right|^{3} }}{3!}} \right],I_{e3} (t) = \mathop {\max }\limits_{t \in [0,1]} \left[ {\frac{{d^{3} I(t)}}{{dt^{3} }} \cdot \frac{{\left| t \right|^{3} }}{3!}} \right] \\ & Q_{e3} (t) = \mathop {\max }\limits_{t \in [0,1]} \left[ {\frac{{d^{3} Q(t)}}{{dt^{3} }} \cdot \frac{{\left| t \right|^{3} }}{3!}} \right],R_{e3} (t) = \mathop {\max }\limits_{t \in [0,1]} \left[ {\frac{{d^{3} R(t)}}{{dt^{3} }} \cdot \frac{{\left| t \right|^{3} }}{3!}} \right] \\ \end{aligned}$$

**Fig. 1:**
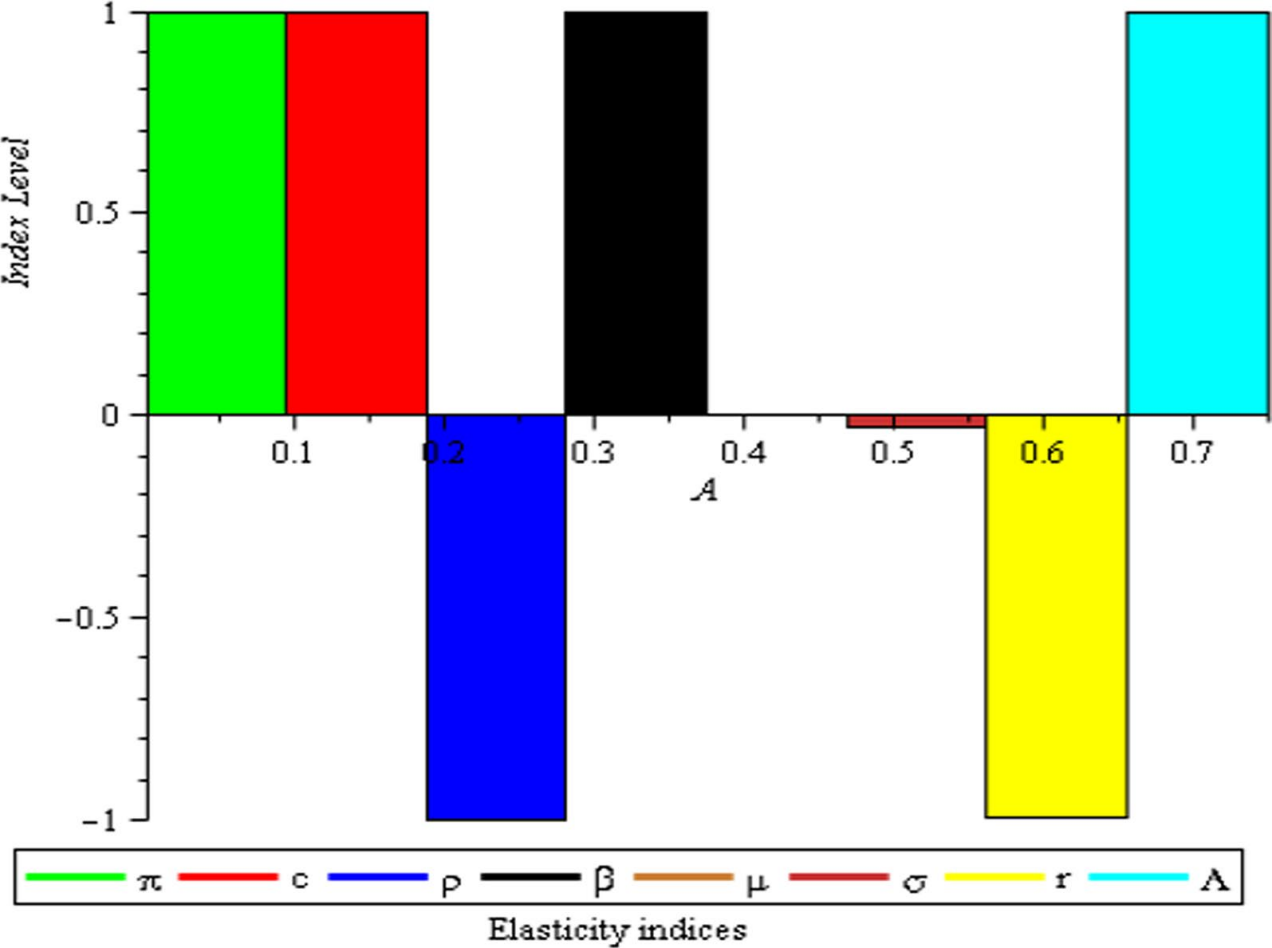
Normalized local sensitivity index of ${\kern 1pt} {\kern 1pt} R_{0}$ on each parameter

The error analysis of the approximate series results demonstrates that the model is validated for actual data simulation since the error generated by the third-order approximation is negligible. We observed that the maximum error is recorded in the susceptible and exposed compartments. This happens because the two classes contain nonlinear variables (Tables 4 and 5; Fig. 1).

**Table 4:** Third-order maximum truncation error for $t \in [0,1]$

$t$	$S_{e3} (t)$	$E_{e3} (t)$	$I_{e3} (t)$	$Q_{e3} (t)$	$R_{e3} (t)$
0	0	0	0	0	0
0.2	0.0009055874376	0.0008941789712	${7}{\text{.605138070}}\, \times \,10^{ - 7}$	0.0002710621426	0.0002711025866
0.4	0.007244699501	0.007153431770	0.000006084110456	0.002168497141	0.002168820693
0.6	0.02445086082	0.02414283222	0.00002053387279	0.007318677851	0.007319769839
0.8	0.05795759601	0.05722745416	0.00004867288365	0.01734797713	0.01735056554
1.0	0.1131984297	0.1117723714	0.00009506422587	0.03388276783	0.03388782333

**Table 5:** Error range of each class at $t \in [0,1]$

$S_{e3} (t)$	$0 \le E_{r} \le 10^{ - 1}$
$E_{e3} (t)$	$0 \le E_{r} \le 10^{ - 1}$
$I_{e3} (t)$	$0 \le E_{r} \le 10^{ - 5}$
$Q_{e3} (t)$	$0 \le E_{r} \le 10^{ - 2}$
$R_{e3} (t)$	$0 \le E_{r} \le 10^{ - 2}$

### Numerical simulation

**Fig. 2:**
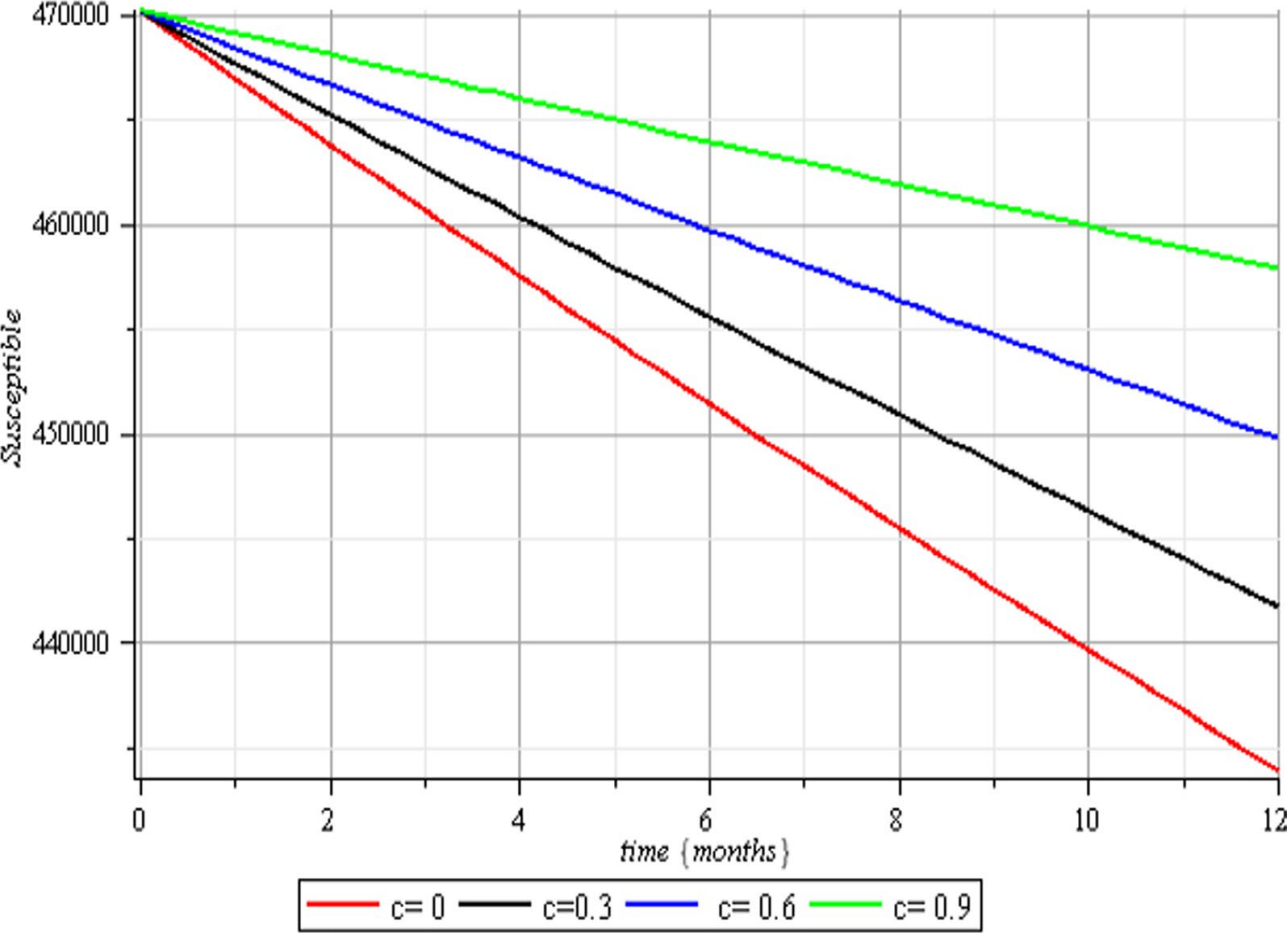
Effects of human submission to physical limitation on the susceptible class

**Fig. 3:**
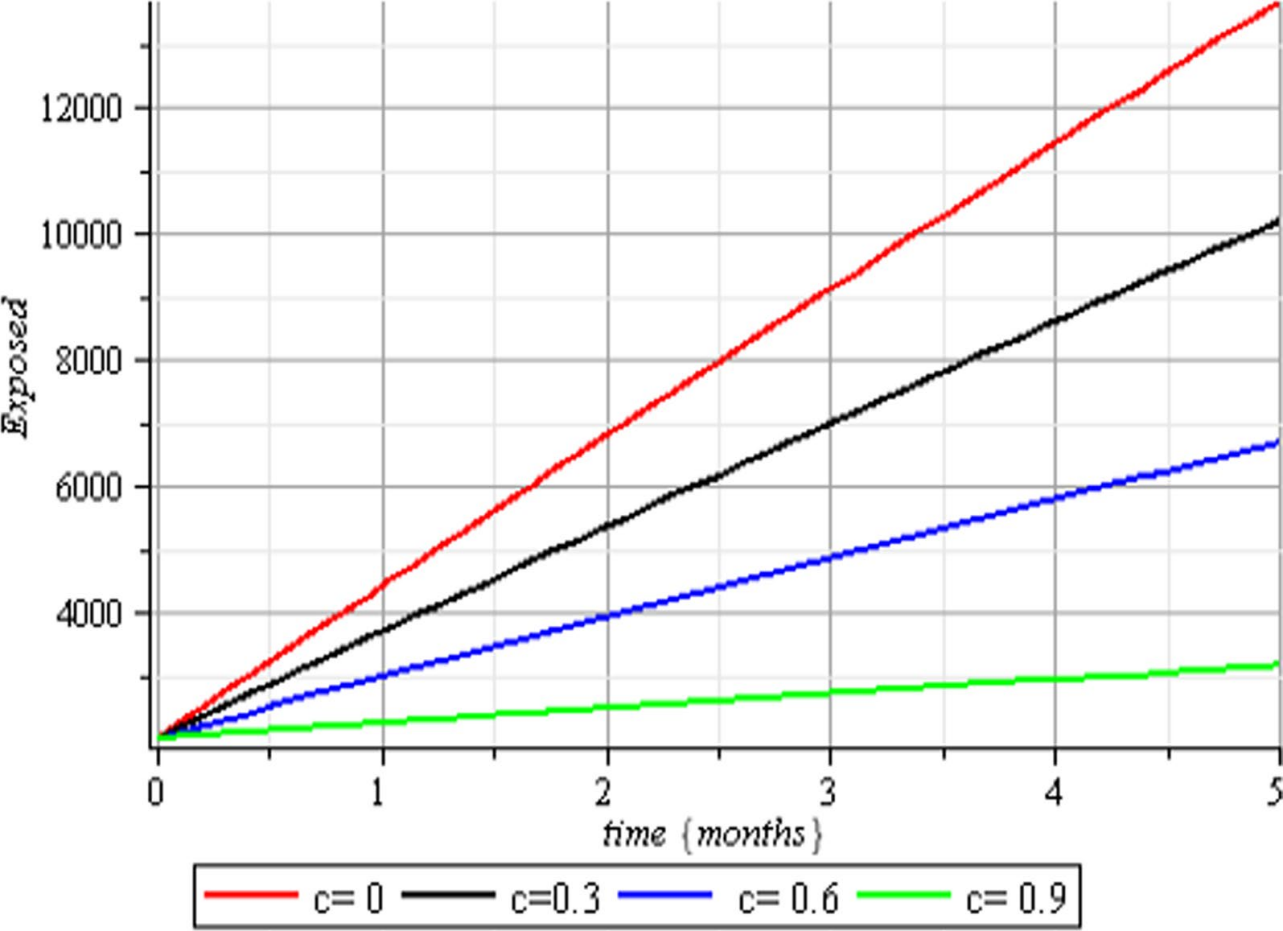
Effect of human submission to physical limitation on the exposed class

**Fig. 4:**
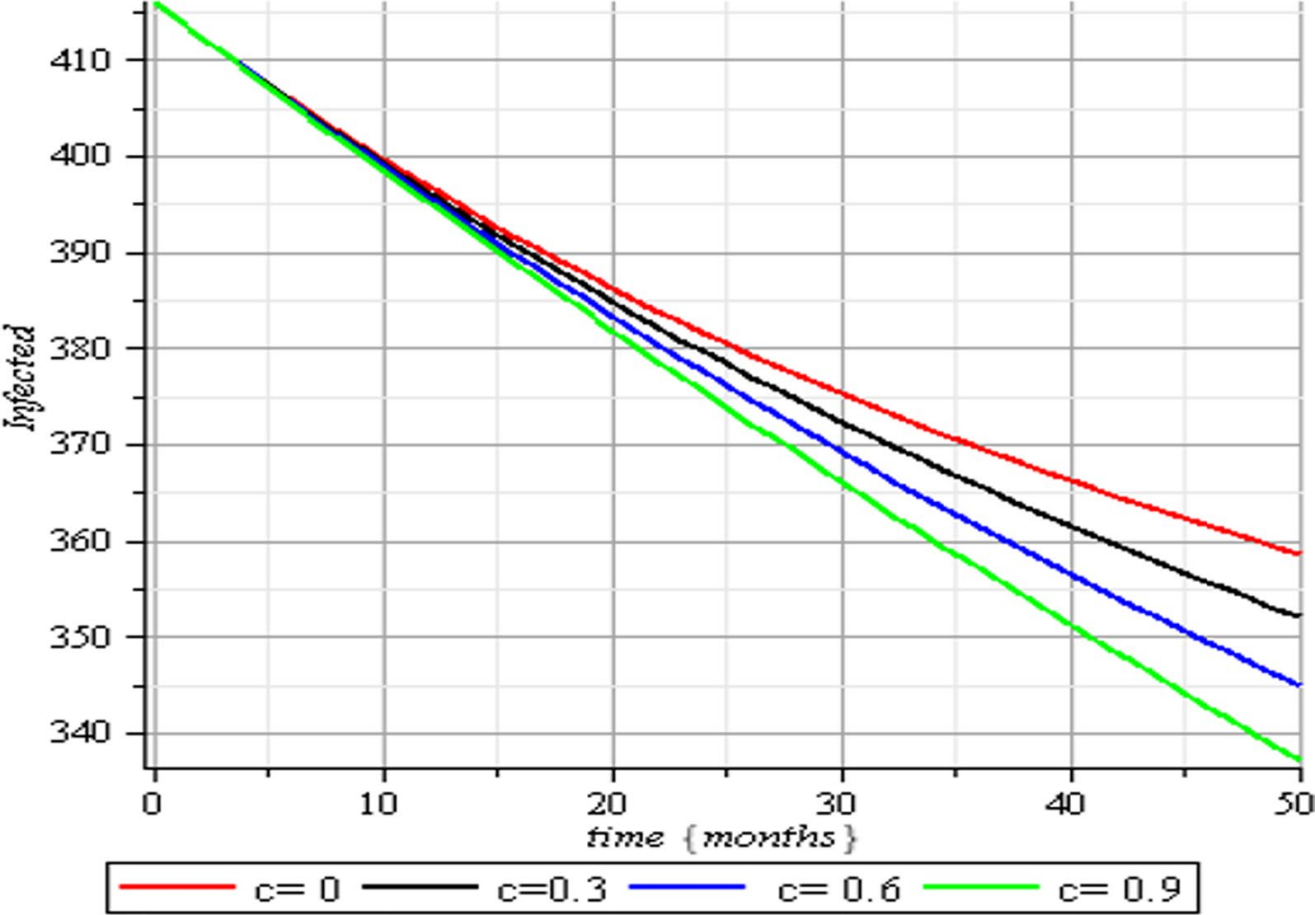
Effects of human submission to physical limitation on the infected class

**Fig. 5:**
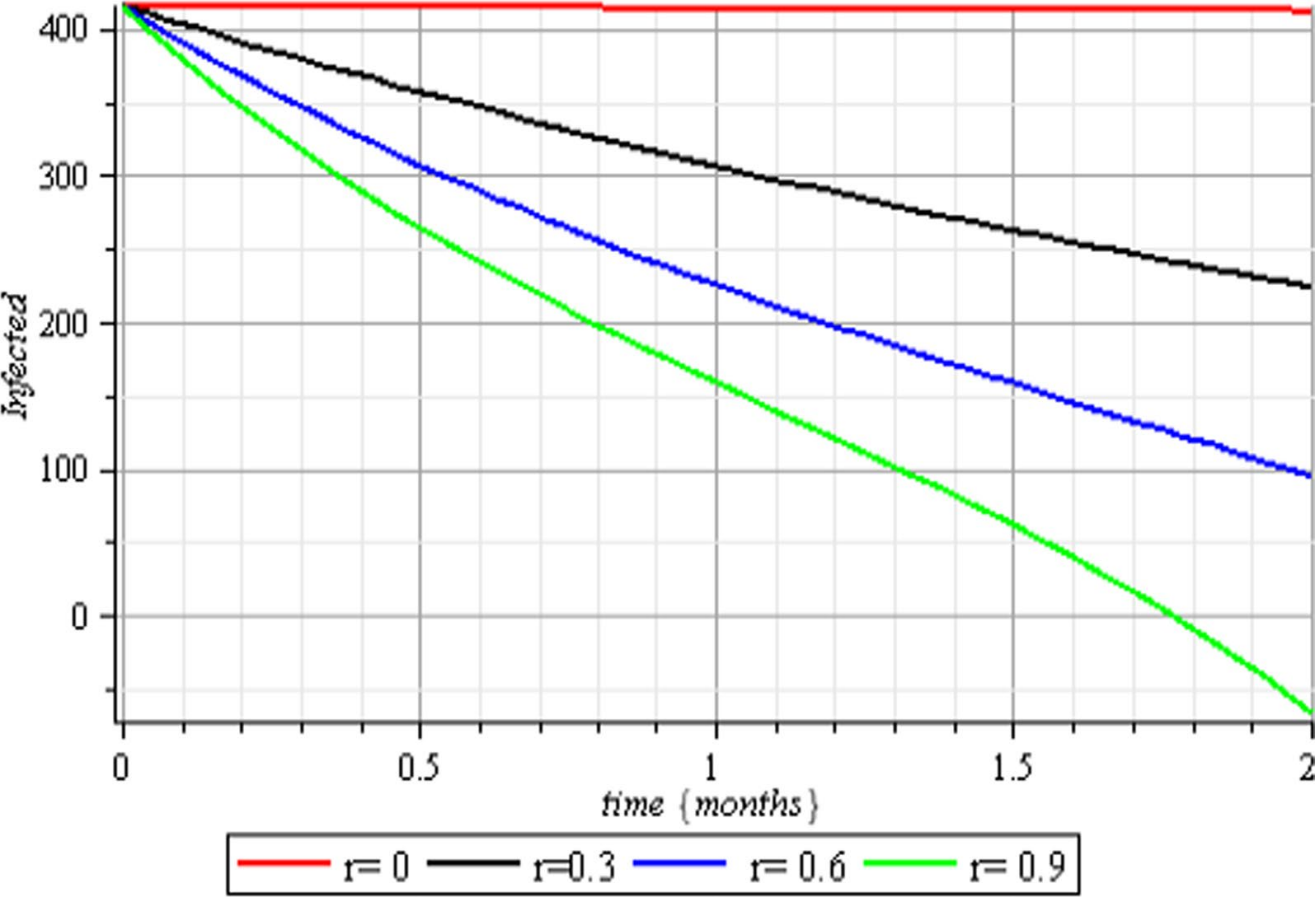
Therapeutic effects on the infected class

**Fig. 6:**
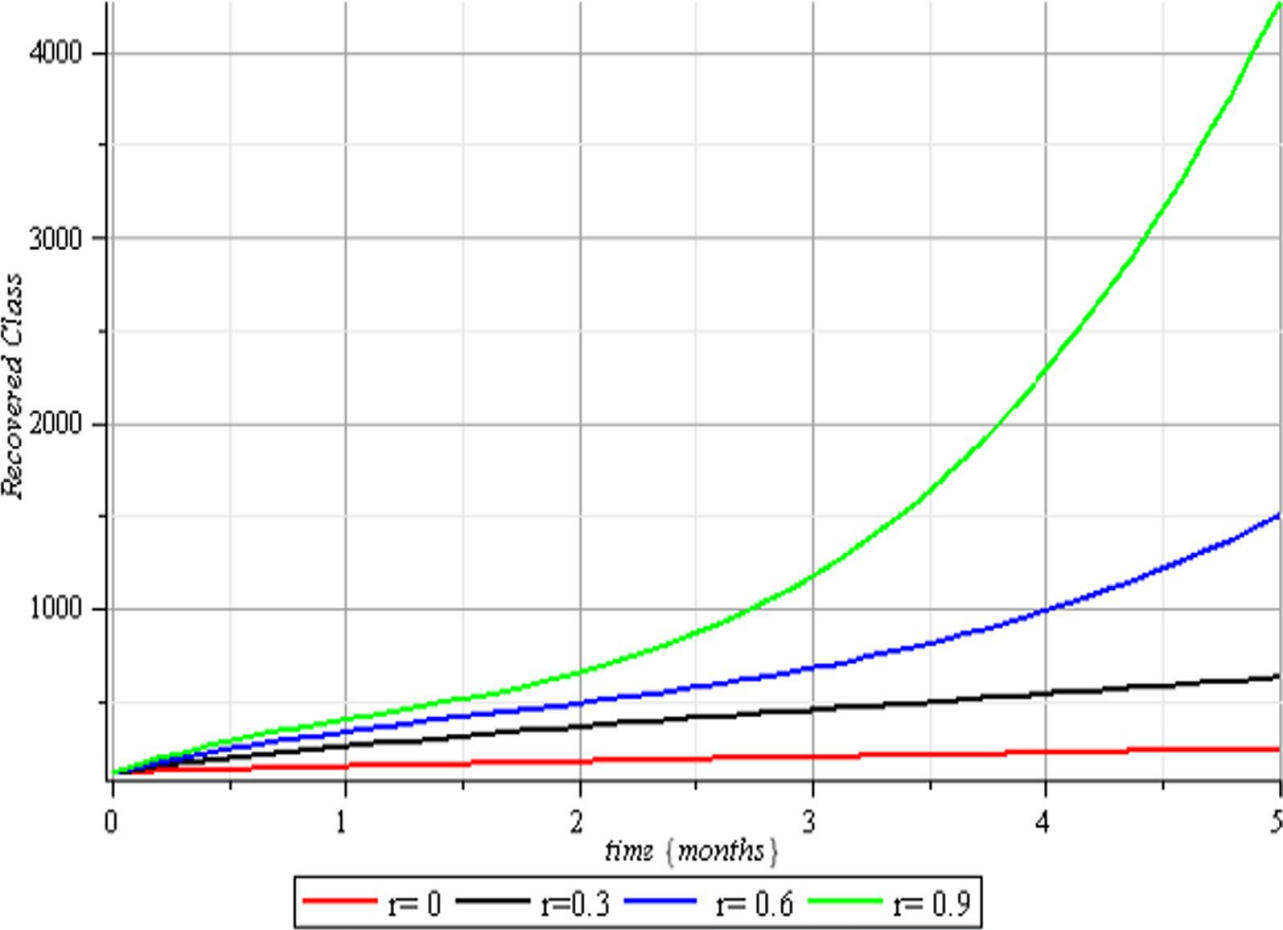
Therapeutic effects on the recovered class

**Fig. 7:**
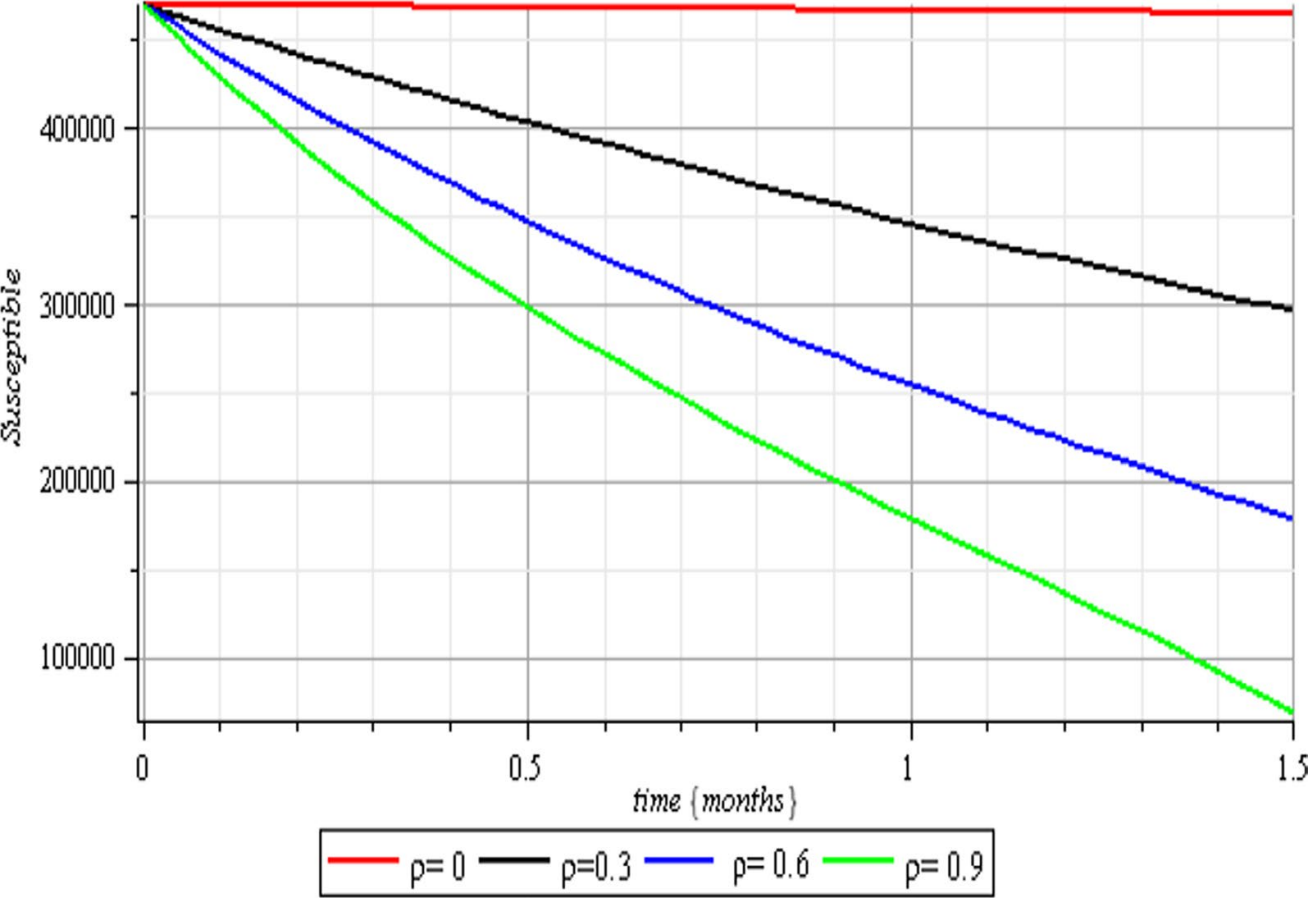
Vaccination effects on the susceptible class

**Fig. 8:**
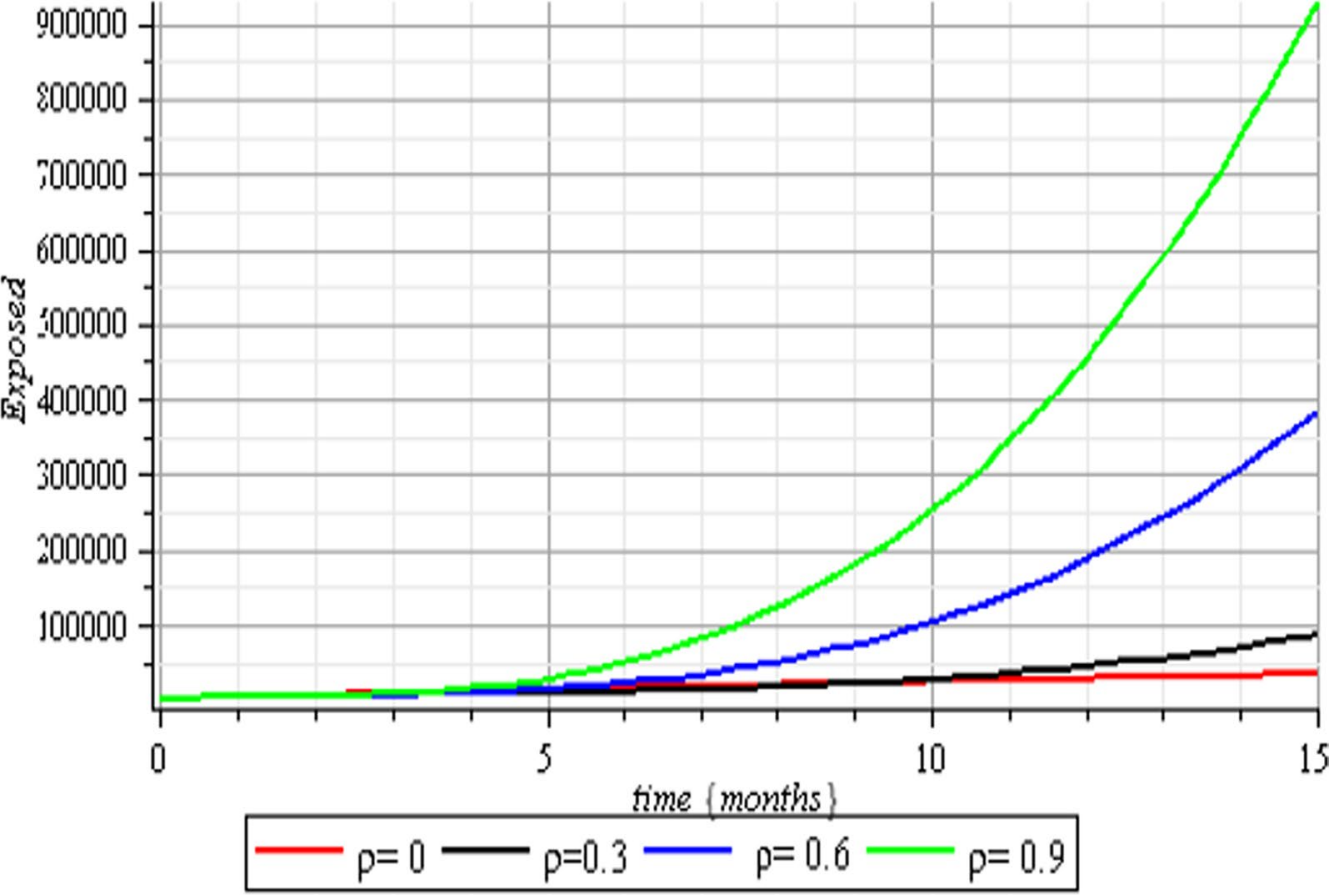
Vaccination effects on the exposed class

**Fig. 9:**
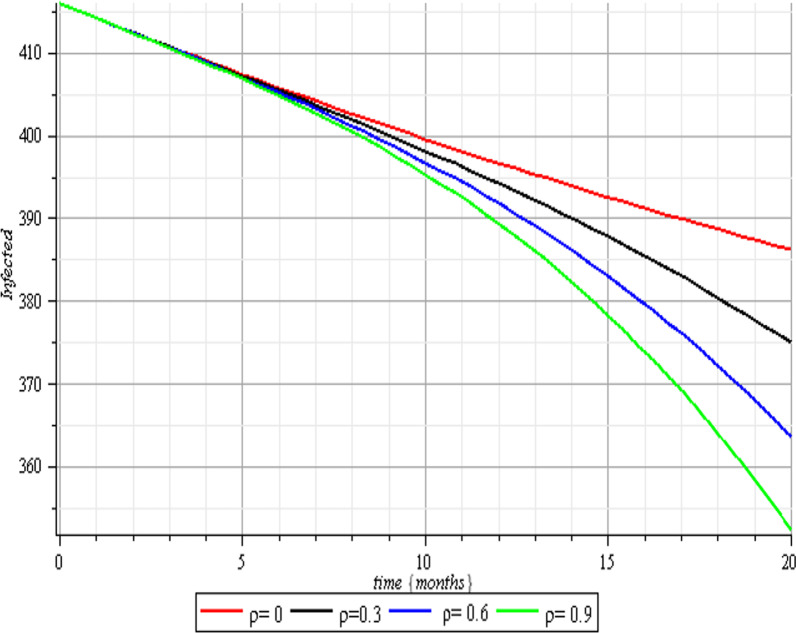
Vaccination effects on the infected class

**Fig. 10:**
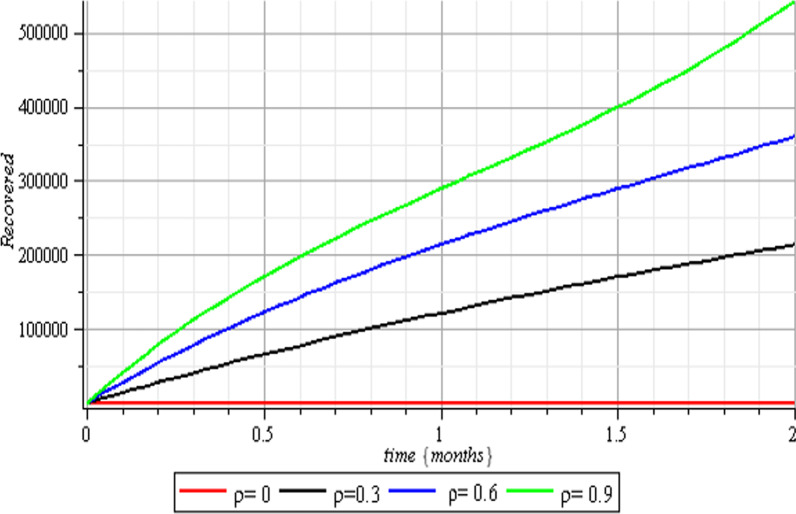
Vaccination effects on the recovered class

In this section, we execute numerical simulations of the mathematical model to rigorously investigate the influence of the incorporated parameters $$\rho ,\,\,c\,\,\& {\text{r}}$$ on the interval $$[0,{1)}$$ to ensure the effectiveness of the regulatory factors integrated in the research. Figures 2, 3, 4, 5, 6, 7, 8, 9, and 10 depict the simulation process's outcomes graphically. Figure 12 reveals the various responses of the exposed and infected human population to combine the influence of the incorporated control parameters.

### Validity of the model

**Fig. 11:**
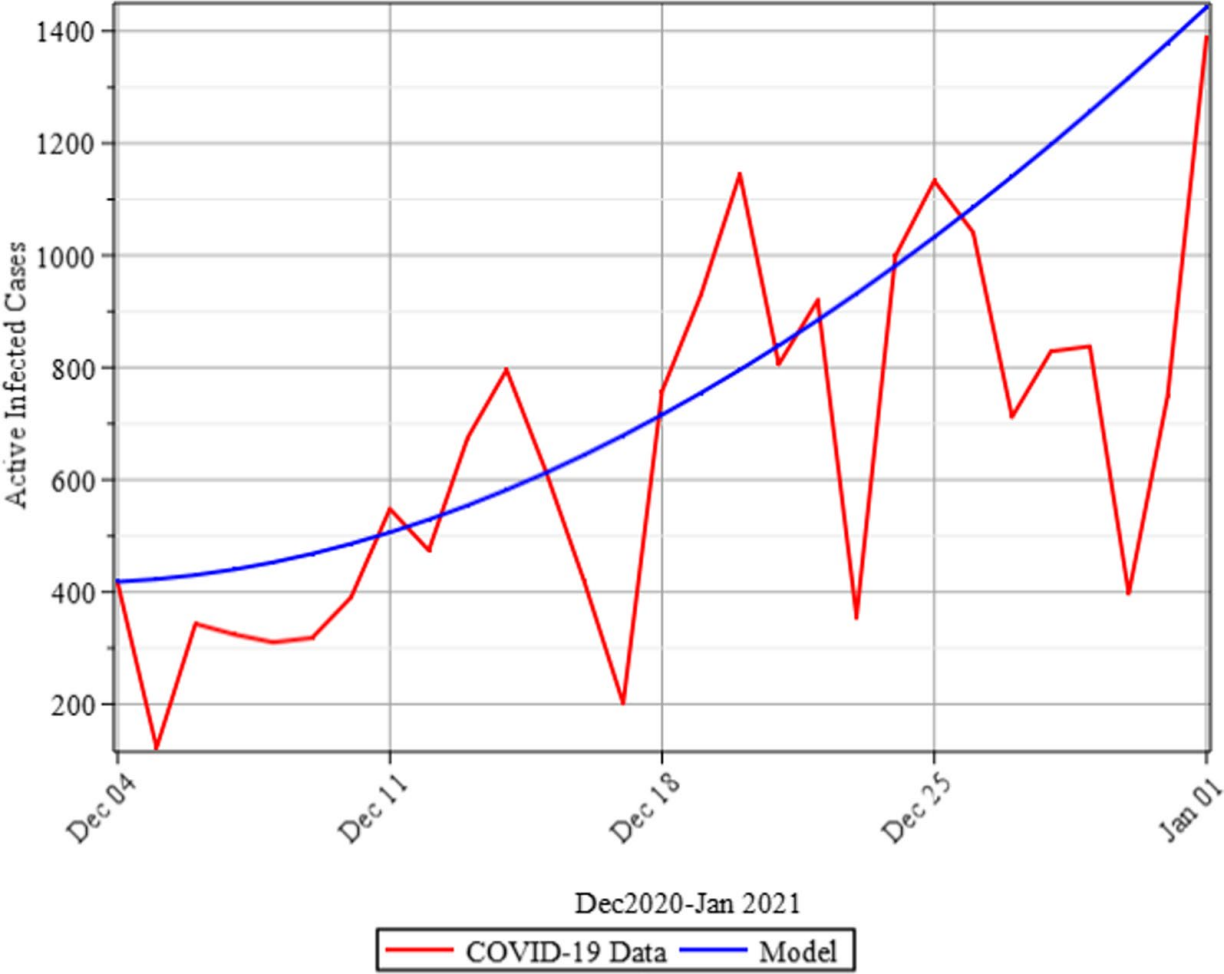
Comparison plots of real-life COVID-19 data and the model’s simulation result

In this section, we assess the model's validity by numerically projecting active infected cases for 30 days using the baseline parameters given in Table 2 and comparing the outcome with a time series plot of daily data of COVID-19 reported by the Nigeria Centre for Disease Control between December 1 and 31, 2020 [21]. The model's validity is evidenced by the fact that it achieves its intended objective. The validation result of the proposed model in Fig. 11 demonstrates that the proliferation in active infected cases when the study's incorporated measures are not used correlates with the time series plot of real COVID-19 data.

## Discussion

**Fig. 12:**
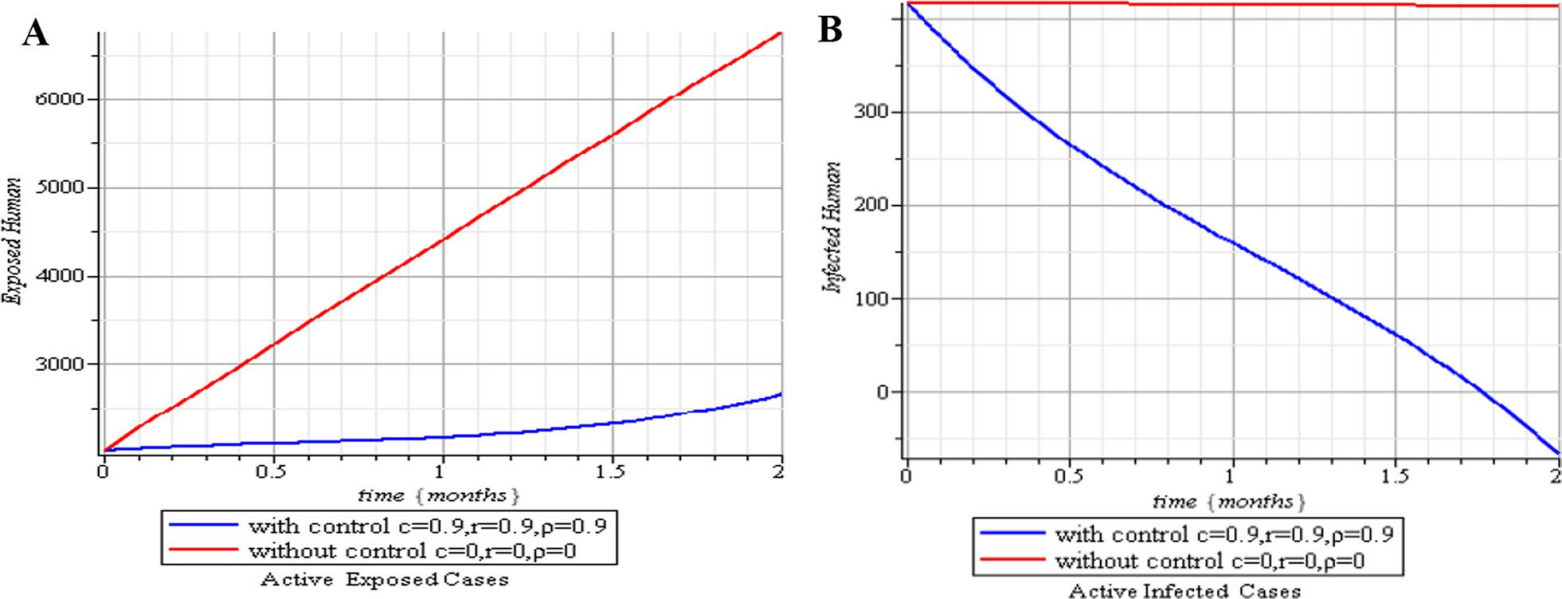
Exposed and infected cases with control and without control

The results of the numerical simulations are discussed in this section. The restriction on population-wide physical contact, extensive imposition of immunization, and the intense care given to the sick patients were all clearly examined and recorded to have increased the number of susceptible and recovered individuals in a COVID-19-enraged population. Remarkably, we observed that the findings of this analysis are consistent with those of earlier research [9], which focused on the impact of piecewise linear therapy on a novel SEIRS model of coronavirus (COVID-19) illness. The maximal imposition of therapy is also included in our study, and it was demonstrated that the count of recovered individuals peaked when therapy is imposed at a maximum level. The simulation results shown in Figs. 5 and 6 revealed that the therapy parameter incorporated in our model exhibits similar responses in recovered and infected patients as obtained earlier in [9]. Hence, therapy is crucial in limiting the transmission of the COVID-19, and we propose that therapy-associated factors such as treatment and medications be effectively levied on infected people in order to increase the number of recovered COVID-19 patients. Figures 2, 3, and 4 examine the impact of physical limitation compliance “c” on the SEIR class. In a similar but distinct study reported in [20], raising the probability of an individual's positive reaction to the curfew and social distancing lowers the basic reproduction number below one, and this will slow the progression of the disease. Figure 2 also shows that limiting physical contact between susceptible and infected people significantly reduces the number of vulnerable people. It can be demonstrated that when the curfew parameter is set to the maximum, there is no infection transmission between the two groups and no progression from susceptible to exposed and infected, indicating that system (1) will eventually approach asymptotically stable disease-free point. Figures 3 and 4 assert that restricting physical contact would substantially lower the number of exposed and sick persons; hence, if the masses failed to comply with the rule of curfew, social distancing, etc., system (1)'s trajectory may approach the endemic point, and all individuals in the recovered class will be wiped out. Although increasing the response rate of an individual's body contact in a disease-manifested system cannot totally prevent the disease's spread, it can reduce it to a bare minimum; hence, we explore the effect of vaccination in reducing infectious disease transmission. Figures 7, 8, 9, and 10 depict the response rates of susceptible, exposed, infected, and recovered population systems to immunization. As revealed in an earlier study [8], immunizing vulnerable individuals minimizes the number of exposed and sick people. Vaccination also showed significant effects in limiting infection propagation in the current study, as recovered individuals were observed to peak when vaccination was fully provided. As a consequence, the disease will be eradicated by the proposed system. The collective impact of the three control mechanisms on the mathematical model is depicted in Fig. 12. In the absence of the three control factors in Fig. 12, it was observed that active cases of exposure and infections of COVID-19 keep projecting. Contrarily, this increasing population of the two classes was observed to be drastically lowered to minimum when the control mechanisms are utilized. Also, the homotopy perturbation approach used in this work has been used to analytically solve and mathematically explore a suggested smoking model utilizing the Caputo–Fabrizio operator in [27]. The approach also effectively generated the solution of our proposed mathematical model since it yielded a stable result which unconditionally describes the dynamics of coronavirus disease, as seen in our simulation results. If time is increased, as seen in several of the graphs, negative population figures will be recorded. An identical dynamic was identified in [28], when the Laplace–Adomian decomposition approach was used to analyze the effect of fractional-order on a smoking model. Because of the small data sets employed, a short time interval was used to prevent a negative population of classes, which would be unrealistic; thus, we propose that for a longer time interval, big values of the initial data should be used so that the concerned population remains positive. Furthermore, since there are several factors associated with the dynamics of disease transmission during pandemic, short time intervals should be considered for authentic results due to different sensitivities of the model parameters to time.

## Conclusions

Epidemic infections, such as COVID-19, cannot be totally eradicated by a single factor. The objectives of this research were achieved as the numerical simulation analysis unconditionally showed the dynamics of COVID-19 spread under different control situations. We also showed that the combined application of vaccine, therapy, and human compliance factors to COVID-19 curfew and social distancing rules has an appreciable effect in stopping the disease's global existence. The mathematical model’s validity and error analysis showed that the model could be applied for further studies on coronavirus disease as it depicts the behavior of real-life COVID-19 situations.

To eradicate the COVID-19 virus, we recommend that governments around the world give immunizations to their vulnerable populations on high range. Furthermore, hospitals should provide superior health-care facilities as well as advanced life-saving technology capable of treating sick people flawlessly. Furthermore, it is critical to underline that governments' efforts to restrict the transmission of the virus can only yield beneficial results if the populace has a receptive attitude toward avoiding disease contact. To constantly eliminate the disease's existence, it is thus recommended that everyone obey curfew, isolation, and the use of nose masks, among other things.

### Recommendations

Although this study demonstrated the diverse reactions of an endangered population to therapy, physical contact limitation, and immunization in a COVID-19-exhibited environment, future research could incorporate introduction of second-stage immunization strategy as well as incidence rate capturing fear of disease transmission. Also, fractional-order analysis of the current mathematical model employing fractional operators like Caputo [29], Caputo–Fabrizio [30], or Atangana–Baleanu [31] might also be useful in examining the disease's dynamical trend on real data.

## Data Availability

All data generated and analyzed during this study are well cited and included in this article.

## Abbreviations

SEIQR: Susceptible, Exposed, Infected, Quarantined, Recovered
SEIRS: Susceptible, Exposed, Infected, Recovered, Susceptible
EIAV: Equine infectious anemia virus
DFE: Disease-free equilibrium
WHO: World Health Organization
NCDC: Nigeria Center for Disease Control
